# Second edition of the recommendations from the Colombian consensus committee for the management of traumatic brain injury in the prehospital setting, emergency department, surgery, and intensive care (Beyond one option for treatment of traumatic brain injury: A stratified protocol [BOOTStraP])

**DOI:** 10.1016/j.bas.2026.106046

**Published:** 2026-04-12

**Authors:** Andrés M. Rubiano, Sandra Lorena Olaya, Christian O. Ramírez, Enoc Noscue Montealegre, Kelly Johanna Arias Osorio, Jonathan Pardo Carranza, Julio César Diez Sepúlveda, Alfonso Bustamante Cristancho, Dumar Javier Figueredo Sanabria, José Luis Castillo García, Juan Diego Ciro, Carlos Eduardo Rebolledo Maldonado, Berhioska Valentina Pérez Velásquez, Adrián Felipe Zapata Lopera, William Ricardo Vargas Escamilla, Carlos Felipe Salgado Bello, Johanna Cecilia Valdeblanquez Atencio, Wendy González, Andrés Salazar, Santiago Cardona-Collazos, Laura M. Loaiza-Cardona

**Affiliations:** a NIHR Global Health Research Group in Neurotrauma, University of Cambridge, UK/Neurosciences Institute, Universidad El Bosque, Bogotá, Colombia/Meditech Foundation, Cali, Colombia; b MEDITECH Foundation, Cali, Colombia; c Colombian Association of Trauma and Emergency Medical Systems (ACTSEM), Colombia; d Sabaneta Volunteer Fire Department (CBVS), Sabaneta, Antioquia, Colombia; e Colombian Association of Emergency Medicine Specialists (ACEM), Colombia; f Vallesalud IPS, Cali, Colombia; g Colombian Association of Critical Care and Intensive Care Medicine (AMCI), Colombia; h Colombian Association of Neurosurgery, Colombia; i Clinica Colombia, Cali, Colombia; j Servicio de Emergencias Medicas, Aeropuerto El Dorado, Bogota, Colombia; k Centro Regulador de Urgencias, Neiva, Colombia; l Fundación Valle del Lili, Cali, Colombia; m Clínica Imbanaco, Cali, Colombia; n Oficina de Gestion de Riesgo, Chía, Colombia; o Clínica Las Americas, Medellin, Colombia; p Clinica Iberoamerica, Barranquilla, Colombia; q Clinica La Maria, Medellin, Colombia; r Hospital Universitario del Valle, Cali, Colombia; s Clinica CEDES, Riohacha, Colombia; t Universidad del Valle, Cali, Colombia

**Keywords:** Traumatic brain injury, Critical care, Emergency care, Prehospital care, Resource-limited settings, Practice guideline

## Abstract

**Introduction:**

Traumatic brain injury (TBI) remains a leading cause of mortality and disability in Colombia and other low- and middle-income countries (LMICs), where disparities in resource availability limit implementation of standardized care. The first edition of BOOTStraP (Beyond One Option for Treatment of Traumatic Brain Injury: A Stratified Protocol), published in 2020, provided guidance for TBI management across variable healthcare settings. Advances in neurotrauma care prompted development of an updated protocol.

**Research question:**

How can TBI management be optimized across prehospital care, emergency care, neurosurgical surgery, and intensive care phases in settings with varying resource availability?

**Material and methods:**

A multidisciplinary panel of 17 national experts in prehospital care, emergency medicine, neurosurgery, and intensive care conducted a structured consensus process using Delphi principles and Nominal Group Technique. Eleven predefined clinical questions were addressed. Recommendations were developed through subgroup work, literature review, and iterative voting, requiring ≥70% agreement at subgroup level and ≥90% plenary consensus. Final algorithms were stratified according to resource availability and healthcare complexity.

**Results:**

The second BOOTStraP edition produced 9 integrated management algorithms covering prehospital transport, emergency care, neurosurgical decision-making, and critical care. Interventions are stratified and color-coded by resource requirements, enabling feasible actions in low-, intermediate-, and high-resource settings. Algorithms integrate neuroprotection goals, triage criteria, surgical indications, and monitoring strategies.

**Discussion and conclusion:**

BOOTStraP provides a pragmatic, phase-based, resource-sensitive framework bridging evidence-based recommendations with real-world constraints. This approach supports context-adapted decision-making and may reduce secondary brain injury while improving standardization of TBI care in resource-limited environments.

## Highlights

• New stratified TBI protocols for variable resource settings.
• Guides decisions from prehospital to ICU with 9 algorithms.
• Telemedicine integration supports resource-limited centers.
• Simplifies complex protocols with color-coded interventions.
• Multidisciplinary consensus enhances real-world applicability.

## 1 Introduction

Traumatic brain injury (TBI) poses a significant public health challenge worldwide as it affects over 60 million people each year and is associated with high mortality and disability, often affecting young and economically active populations, representing the leading cause of death in people between the ages of 15 to 29 (Dewan et al., 2019; Vos et al., 2015). Road traffic accidents are the leading cause of fatality secondary to TBI worldwide (Dewan et al., 2019).

Epidemiological studies reveal regional variations in TBI incidence and prevalence. Three times more cases occur in rural areas and low—and middle-income countries (LMICs) than in urban areas of high-income countries (HICs) (Mowafi et al., 2019). Disability and mortality rates are also higher in LMICs and rural areas, where 89% of deaths from a traumatic cause occur (Norton et al., 2013). This increased mortality and disability linked to TBI in lower-income regions is related to inadequate prevention, insufficient control of risk factors, and limited capacity for acute care and rehabilitation (Brown et al., 2019).

The World Health Organization's Global Report on Road Safety indicates that 90% of deaths from road traffic accidents (RTAs) occur in LMICs. Although LMICs comprise over 80% of the world's population, they hold just over 50% of the world's registered vehicles, revealing a disproportionate number of road traffic fatalities (World Health Organization, 2023).

Colombia continues to experience a significant incidence of trauma from social violence and traffic accidents. Estimates indicate that the proportion of these traumas related to traumatic brain injury (TBI) can be as high as 70%. Unfortunately, there is limited reliable data available in Colombia regarding deaths attributed to TBI. However, autopsy reports from the National Institute of Legal Medicine and Forensic Sciences suggest that 70% of deaths resulting from violence and 90% of fatalities from road traffic accidents (RTAs) are associated with TBI (Instituto Nacional de Medicina Legal y Ciencias Forenses, 2018; Instituto Nacional de Medicina Legal y Ciencias Forenses, 2024).

In 2014, the Colombian Ministry of Health funded the development of evidence-based clinical practice guidelines (CPGs) for the diagnosis and treatment of severe TBI in adults (Colombia. Ministerio de Salud y Protección Social et al., 2014). This guideline was developed by the Meditech Foundation with the objective of reducing the heterogeneity of care for these patients through scientific evidence and recommendations from expert clinical representatives from multiple disciplines involved in the comprehensive care of TBI patients. During the implementation of this guideline, it was identified that the heterogeneity of resources available for the care of severe TBI was a critical barrier that was not addressed in the CPGs.

To overcome the barrier of heterogeneity in the resources available for the care of patients with TBI in the different phases of treatment, the first version of the stratified protocol entitled Beyond One Option for Treatment of Traumatic Brain Injury: A Stratified Protocol (BOOTStraP) was developed and published in 2020 (Rubiano et al., 2020). This protocol was based on Colombian health services regulations, where complexity levels are described as shown in Table 1. A consensus process involving clinical experts was used to create protocols that articulated treatment options for TBI specific to various levels of resources and complexity across the prehospital, emergency care, neurological surgery, and intensive care phases. The expert panel included representatives from the Colombian Association of Trauma and Emergency Medical Systems, the Colombian Association of Specialists in Emergency Medicine, the Colombian Association of Neurosurgery's Neurotrauma Chapter, and the Colombian Association of Critical Care Medicine and Intensive Care through the Chapter of Neurointensive Care.

**Table 1:** **Classification of Complexity Levels in Prehospital, Emergency, Neurosurgical, and Intensive Care Services.** AED = automated external defibrillator; CC = critical care; CT = computed tomography; ICU = intensive care unit. *Source of definitions:* Colombian Ministry of Health Technical Documents and World Health Organization Technical Documents (Ministerio de Salud Y Protección Social de Colombia, 2019; Mock, 2004).

Level of Resources							
Prehospital Care		Emergency Care		Neurological Surgery		Intensive Care	
Basic Emergency Transport	Advanced Emergency Transport	Basic Health Facility (without CT) Low Complexity	Advanced Health Facility (with CT) Medium-High Complexity	Operating Room with CT Access But Without Neurological Surgery Capacity	Operating Room with CT Access And Neurological Surgery Capacity	ICU with CT in a center of Medium Complexity	ICU with CT in a center of Medium-High Complexity
Vehicle with a first responder (with or without training). Vehicle with or without electronic monitoring of vital signs. Vehicle without advanced airway management equipment. Vehicle with or without IV fluid infusion capability.	Vehicle with: Physician, nurse, or EMT. Driver with training in BLS. Mechanical ventilator with battery for at least 4 h. Electronic vital signs monitor. AED. Advanced airway management kit. Medications for ALS.	Facility with: General physician with ALS training. Nurse or nurse technician with BLS training. Electronic vital signs monitor. AED. Cardiac arrest kit. Oxygen. Drug infusion pumps. Airway suction system. Basic radiology without CT. Cristalloid fluids. Pharmacological support. Basic clinical laboratory.	Facility with: General physician, emergency medicine specialist, or family physician. Available consults from general surgery, internal medicine, and pediatrics. Clinical laboratory. Radiology service with CT. Pharmacy. Respiratory therapy. Blood transfusion. Health care transport. Operating room with access to anesthesiology services	Facility with: Anesthesiologist. General surgeon. Surgical instrumentation. Surgical nurses. Clinical laboratory. Pharmacy. Basic surgical equipment. No access to neurosurgical care.	Facility with: Anesthesiologist. Surgical instrumentation. Surgical nurses. Clinical laboratory. Pharmacy. Advanced surgical equipment. Access to neurosurgical care.	Facility with: Respiratory therapist. Electronic vital signs monitor. Cardiac arrest kit. Advanced airway management equipment. Advanced drug management for pain and vasoactive drugs. Specialized unit for critically ill patients, staffed either by a CC specialist or, in their absence, by a general physician with ICU training.	Facility with: CC specialist. CC trained respiratory therapist. Mechanical ventilator. Cardiac arrest kit. Advanced airway management equipment. Electronic vital signs monitor. Neurosurgery consultation is available. CT and MRI are available. Full spectrum of specialists for consultation.

Several advances in neurotrauma care published between 2019 and 2023 prompted the development of this updated protocol using the same Delphi-based methodology. The key changes introduced in the second edition, compared with the first, include: 1. Reduction in the total number of algorithms from 10 to 9, with a simplified and restructured layout to improve usability; 2. integration of non-invasive neuromonitoring tools (optic nerve sheath diameter [ONSD] measurement, automated pupillometry and transcranial Doppler [TCD]) into emergency and intensive care algorithms; 3. Incorporation of serum biomarkers (GFAP and UCH-L1) as adjuncts to triage decision-making; 4. Updated CT interpretation guidance using the ABCDE-Z method, including the Zumkeller Index; 5. Revised and updated pharmacological protocols based on evidence published after the first edition; 6. Explicit telemedicine guidance for resource-limited centers without neurosurgical coverage; 7. Expanded surgical indications table incorporating non-invasive monitoring parameters in decision making; and 8. Updated neuroprotective goals aligned with current international consensus statements This article outlines the methods and recommendations of the second edition of BOOTStraP.

## 2 Materials and methods

Seventeen experts were invited to a consensus meeting to develop an updated protocol for the comprehensive care of adults with TBI. The process was guided by the principles of the Delphi method and the Nominal Group Technique. Participants were organized into subgroups according to their areas of expertise (prehospital care, emergency medicine, neurosurgery, and intensive care) with each subgroup led by an appointed moderator.

One month before the meeting, each subgroup received preparatory materials, including current CPGs and scientific literature relevant to key interventions within their respective domains (Rubiano et al., 2020; Ghaith et al., 2022; Chesnut et al., 2020a; Hawryluk et al., 2019; Wilson et al., 2022; Bossers et al., 2021; Gruen et al., 2020; Godoy et al., 2023a; Zeiler et al., 2022; Ahmadi et al., 2023; Iaccarino et al., 2021; Hutchinson et al., 2019; Shukla, 2020; Hutchinson et al., 2023; Picetti et al., 2023; Papa et al., 2022; Wiegers et al., 2021; Cook et al., 2020; Hawryluk et al., 2020; Messina et al., 2022; Layard et al., 2020; Marchesini et al., 2023; Núñez-Patiño et al., 2020; Pal, 2020; Meyfroidt et al., 2022; De Souza et al., 2021; Khan and Khan, 2020; Agrawal, 2020; Barton et al., 2024; Lulla et al., 2023; The PATCH-Trauma Investigators and the ANZICS Clinical Trials Group, 2023; Sewalt et al., 2021; Smith et al., 2022; Godoy et al., 2023b; Godoy et al., 2023c; Clavijo et al., 2019; Tan et al., 2017; Rasulo et al., 2022; Maas et al., 2022; Griswold et al., 2022; Picetti et al., 2019).

The consensus conference was held in August 2023 in Cali, Colombia. The two-day process began with field-specific panel discussions, during which each subgroup worked independently to draft preliminary protocols based on a set of pre-established questions detailed later in the document. These protocols were refined through a series of internal voting rounds, with a 70% agreement threshold required to approve each recommendation at the subgroup level. Voting was conducted in-person by rising hands during plenary and subgroup sessions. Each individual recommendation item was voted on separately before being integrated into composite algorithms; this ensured that each discrete clinical decision point achieved the required agreement threshold independently. Facilitators from the methodological team recorded all votes and tracked consensus levels throughout the process. Voting results for each recommendation are documented and reported as supplementary material (Supplementary Material S1).

Following this, a representative from each subgroup presented their recommendations to the full assembly. These proposals were then discussed collectively, considering both scientific evidence and expert consensus. Each recommendation underwent additional rounds of discussion and voting, requiring a 90% agreement rate for final endorsement. Throughout the process, facilitators from the methodological team provided continuous support during both the deliberation and voting sessions.

In the final session, the management algorithms that integrated the approved recommendations were presented, tailored, and stratified according to the level of resource availability across centers of varying complexity (Supplementary material S2). The outcome of this process was the second edition of BOOTStraP—Beyond One Option for Treatment of Traumatic Brain Injury: A Stratified Protocol.

The field-specific panel discussions addressed the following questions:

### 2.1 Prehospital Care

1. What is the best up-to-date protocol for managing a patient with a head injury during basic emergency transport, where only essential equipment is available and personnel lack training in advanced interventions?
2. What is the best up-to-date protocol for managing a patient with a head injury during advanced emergency transport, where personnel are fully trained and equipped to perform advanced interventions?

### 2.2 Emergency Care

3. What is the best up-to-date protocol for managing a patient with a head injury in a low-complexity emergency department, where only basic equipment is available and staff lack training in advanced interventions?
4. What is the best up-to-date protocol for managing a patient with a head injury in a medium-complexity emergency department, where intermediate equipment (e.g., Computed Tomography) is available and only some staff are trained in advanced interventions?
5. What is the best up-to-date protocol for managing a patient with a head injury in a high-complexity emergency department, where advanced equipment is available and all staff are fully trained in advanced interventions?

### 2.3 Neurological Surgery

6. What is the best up-to-date protocol for managing a patient with a head injury requiring urgent surgery in a facility without neurosurgical services, but where general surgery and CT imaging are available?
7. What is the best up-to-date protocol for managing a patient with a head injury requiring urgent surgery in a facility with neurosurgical capabilities, but without access to an intensive care unit (ICU)?
8. What is the best up-to-date protocol for managing a patient with a head injury requiring urgent surgery in a fully equipped facility with immediate access to neurosurgery, MRI, and ICU care?

### 2.4 Intensive Care

9. What is the best up-to-date protocol for managing a head injury patient in a postoperative recovery area equipped with only basic monitoring and staffed by personnel without ICU-specific training?
10. What is the best up-to-date protocol for managing a head injury patient in an intermediate care unit, where monitoring equipment is moderately advanced and only some staff have ICU-level training?
11. What is the best up-to-date protocol for managing a head injury patient in a general intensive care unit, where advanced equipment is available and all staff are fully trained in ICU-level care?

## 3 Results

### 3.1 How to interpret and use the proposed algorithms

We established categories for each phase of treatment based on real-world scenarios shared by experts from various regions of the country. These scenarios were also identified through multiple surveys conducted during the development of the Colombian TBI guidelines (Rubiano et al., 2013; Puyana et al., 2014).

● Prehospital Care:
  ○ Basic Emergency Transport.
  ○ Advanced Emergency Transport.
● Emergency Care:
  ○ Low complexity EDs without CT.
  ○ Medium–high complexity EDs with CT.
● Neurological Surgery:
  ○ Centers without access to neurosurgical services but with general surgical capabilities.
  ○ Centers equipped with neurosurgical services but no ICU capacity.
  ○ Centers with both neurosurgical services and ICU capabilities.
● Intensive Care:
  ○ Centers that lack dedicated intensive care units but can provide basic postoperative monitoring in a recovery room setting.
  ○ Centers that do not have full ICU capabilities but can offer an intermediate level of care with partial monitoring and limited critical care support.
  ○ Centers with a fully functional intensive care unit, capable of delivering comprehensive critical care and continuous monitoring.

**Fig. 1:**
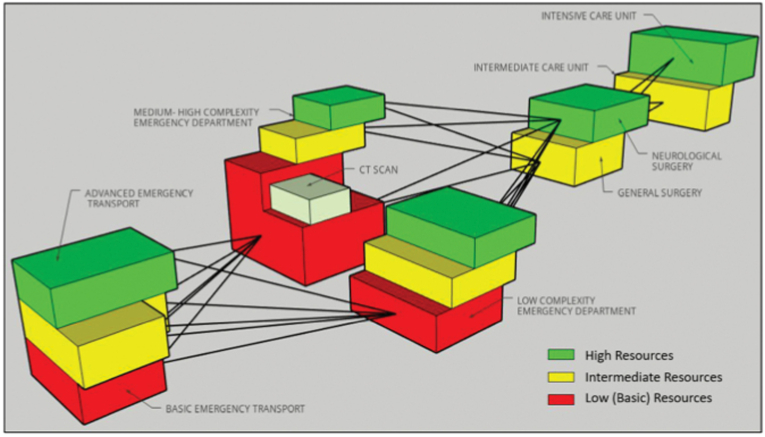
Three-dimensional stratified scheme according to the level of resources and complexity.

For each scenario, treatment algorithms were created. These algorithms are not organized by rigid categorizations but are instead stratified. This approach allows healthcare providers operating within any of the four treatment phases to select the most appropriate intervention based on the resources available to them, as illustrated in Fig. 1.

In the algorithms, treatment options are color-coded to reflect the level of resource availability required for implementation:

- Red indicates that the intervention can be performed in settings with the lowest level of resources.
- Yellow represents interventions suitable for facilities with an intermediate level of resources.
- Green signifies interventions typically available in the most advanced care settings.

All proposed interventions within the algorithms are adaptable and can be selected and applied according to the specific resources available at each level of care. These algorithms are designed as clinical decision-support tools, not prescriptive protocols to be followed mechanically. Each recommendation must be interpreted in the context of the individual patient's clinical status, comorbidities, and the evolving clinical situation. Providers are expected to exercise independent clinical judgment and deviate from the suggested pathway when the patient's condition warrants it. The algorithms do not replace clinical expertise and should be used in conjunction with, not as a substitute for, individualized patient assessment.

#### 3.1.1 Prehospital care

##### 3.1.1.1 Question 1

● What is the best up-to-date protocol for managing a patient with a head injury during basic emergency transport, where only essential equipment is available and personnel lack training in advanced interventions?

##### 3.1.1.2 Recommendations

Patients with mild head injury (GCS 13-15) who do not meet high-risk clinical criteria (Table 2) may be transported using a Basic Emergency Transport (BET) unit, if they are clinically stable and the estimated transfer time is less than 30 min. If the transfer time exceeds 30 min or the patient is unstable and Advanced Emergency Transport (AET) is available, it should be preferred to ensure appropriate monitoring and timely intervention.

**Table 2:** **Criteria for the transfer of TBI patients to high-complexity facilities with access to neuroimaging and neurosurgical consultation.** TBI = Traumatic Brain Injury; GCS = Glasgow Coma Scale.

It is recommended that patients with moderate to severe TBI (GCS 3-12) should be transferred immediately to high-level care hospitals with access to neuroimaging and neurosurgery.
It is recommended that patients with mild TBI (GCS 13-15) who present one or more of the following criteria be referred for evaluation at an institution that has access to neuroimaging and neurosurgery:
GCS <15 up to 2 h after injury
Severe headache
Two or more episodes of vomiting
Skull fracture, including depressed fractures or clinical signs of skull base fractures such as raccoon eyes, retro auricular ecchymosis, otorrhea and rhinorrhea
Age ≥60 years
Blurred vision or diplopia
Posttraumatic seizure
Focal neurologic deficit
Previous craniotomy
Fall from height ≥1.5 m
Retrograde amnesia ≥30 min or anterograde amnesia
Suspected intoxication from alcohol or other psychoactive substances
Use of anticoagulants, antiplatelet drugs or known coagulopathies
Pregnancy
Penetrating TBI

**Fig. 2:**
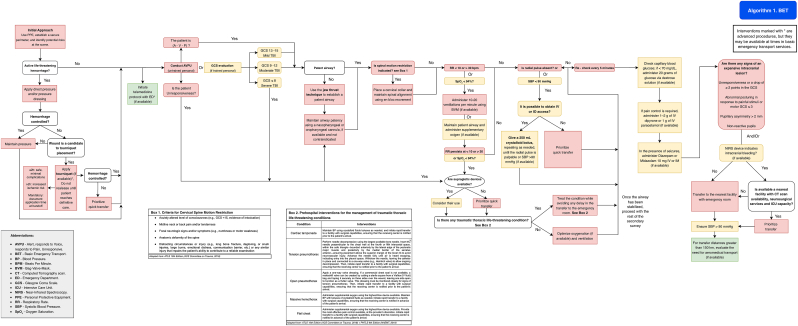
Management algorithm for patients with traumatic brain injury in basic emergency transport (algorithm 1).

The use of BET is contraindicated in cases of moderate to severe traumatic brain injury. If BET is the only available option, see Fig. 2 (Algorithm 1).

In all cases, a sequential and structured approach should be followed, with priority given to the initial stabilization of the patient and the prevention of secondary brain injury, as outlined in Fig. 2 (Algorithm 1), Supplementary material S3 and Table 3, Table 4, without delaying on-scene time to more than 30 min.

**Table 3:** **Criteria for Cervical Spine Motion Restriction.***From: National Association of EMS Physicians. EMS spinal precautions and the use of the long backboard – A joint position statement of the National Association of EMS Physicians and the American College of Surgeons Committee on Trauma. 2018* (Fischer et al., 2018).

● Acutely altered level of consciousness (e.g., GCS <15, evidence of intoxication)
● Midline neck or back pain and/or tenderness
● Focal neurologic signs and/or symptoms (e.g., numbness or motor weakness)
● Anatomic deformity of the spine
● Distracting circumstances or injury (e.g., long bone fracture, degloving, or crush injuries, large burns, emotional distress, communication barrier, etc.) or any similar injury that impairs the patient's ability to contribute to a reliable examination

**Table 4:** Advanced airway management protocol.

Medication	Option 1	Option 2	Option 3
Inductors	Ketamine Amp x 500 mg Dose: 1.5 - 2 mg/kg For a patient of 70 kg: 105-140 mg	Etomidate Amp x 20 mg Dose: 0.3 mg/kg For a patient of 70 kg: 21 mg	Midazolam Amp x 5 mg/amp x 15 mg Dose: 0.1-0.3 mg/kg For a patient of 70 kg: 7-21 mg
Muscular blockers	Succinylcholine Amp x 250 mg Dose: 1 - 2 mg/kg For a patient of 70 kg: 70-140 mg	Rocuronium Amp x 50 mg Dose: 0.7 - 1 mg/kg For a patient of 70 kg: 50-70 mg	Vecuronium Amp x 50 mg Dose: 0.1 mg/kg For a patient of 70 kg: 7 mg
Analgesics	Fentanyl Amp x 500 μg Dose: 2 - 4 μg/kg For a patient of 70 kg: 140 -280 μg	Ketamine Amp x 500 mg Dose: 1.5 - 2 mg/kg For a patient of 70 kg: 105-140 mg	

**Note:** select any option for each one of the categories according to the availability of medications.

**Fig. 3:**
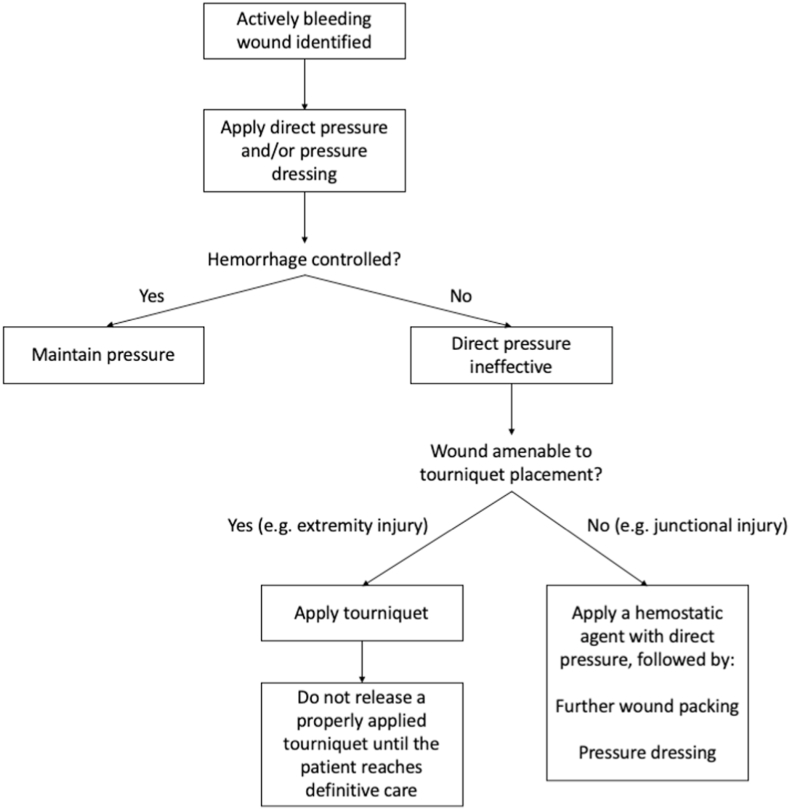
Hemorrhage control protocol.

When a tourniquet is applied for extremity hemorrhage control in this scenario, the time of application must be clearly documented. Current evidence supports that tourniquets can be safely applied for up to 2 h with minimal risk of complications; beyond this threshold, the risk of limb ischemia and systemic complications increases significantly, and tourniquet times exceeding 4 h are associated with reduced limb salvage rates and higher mortality (Fig. 3). The receiving team must be notified of the application time at handoff to guide decisions regarding assessment and removal at a facility with surgical capability (Joarder et al., 2023).

##### 3.1.1.3 Question 2

● What is the best up-to-date protocol for managing a patient with a head injury during advanced emergency transport, where personnel are fully trained and equipped to perform advanced interventions?

##### 3.1.1.4 Recommendations

**Fig. 4:**
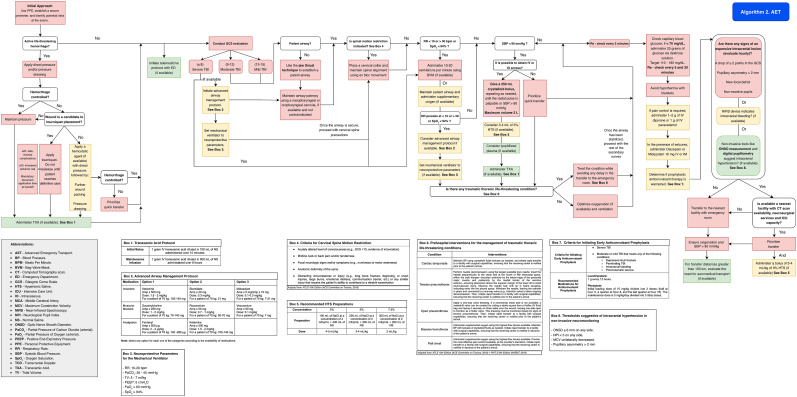
Management algorithm for patients with traumatic brain injury in advanced emergency transport (algorithm 2).

It is recommended that adult patients with head injury be managed according to Fig. 4 (Algorithm 2) when Advanced Emergency Transport (AET) is available.

If the Glasgow Coma Scale (GCS) score is ≤ 8 or there is evidence of airway compromise, airway-clearing maneuvers should be performed, and advanced airway management should be considered, as detailed in Table 4.

On-scene time should be limited to no more than 30 min to reduce delays in definitive care.

#### 3.1.2 Emergency care

##### 3.1.2.1 Question 3

● What is the best up-to-date protocol for managing a patient with a head injury in a low-complexity emergency department, where only basic equipment is available and staff lack training in advanced interventions?

##### 3.1.2.2 Recommendations

**Fig. 5:**
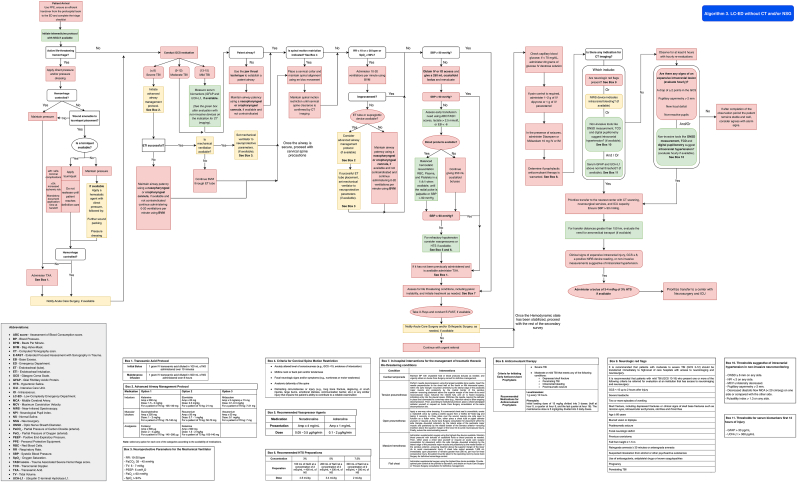
Management algorithm for patients with traumatic brain injury in low-complexity EDs without CT (algorithm 3).

Patients with traumatic brain injury (TBI) presenting to low-complexity emergency departments, where equipment is limited and staff are not trained in advanced management, should be treated following a sequential approach as outlined in Fig. 5 (Algorithm 3) and Supplementary material S3 and Table 2, Table 3, Table 4, Table 5, Table 6, Table 7, Table 8, Table 9, Table 10, Table 11, Table 12.

**Table 5:** Criteria for advanced airway interventions.

Advanced Airway Management Protocol	
**Respiratory rate**	<10 bpm of >30 bpm
**SpO_2_/FiO_2_ Ratio**	<160
**Arterial gasometry findings**	pH < 7.2 or pH < 7.3 associated with respiratory fatigue
**PaO_2_/FiO_2_ Ratio**	<100 despite optimal oxygen therapy
**Hemodynamic instability**	Grade III-IV Shock
**GCS**	≤8 or GCS deterioration ≥2 points + Surgical indication
**Maxillofacial fractures**	Aspiration risk
**Neck injuries**	Expanding neck hematomas, tracheal or laryngeal injuries, inhalation injuries from associated burns, concurrent facial burns, stridor, or voice changes

**Table 6:** **Tranexamic Acid Protocol.** Administration is only recommended within 3 h following trauma. NS = Normal Saline.

Tranexamic Acid Protocol	
**Initial Bolus**	1 g IV tranexamic acid diluted in 100 mL of NS administered over 10 min
**Maintenance Infusion**	1 g IV tranexamic acid diluted in 500 mL of NS administered over 8 h

**Table 7:** Recommended vasopressor agents.

Vasopressor Agents		
**Medication**	Noradrenaline	Adrenaline
**Presentation**	Amp x 4 mg/mL	Amp x 1 mg/mL
**Dose**	0.05 - 0.5 μg/kg/min	0.1 - 2 μg/kg/min

**Table 8:** **Recommended HTS Preparations.** All concentrations mentioned above can initially be administered via a peripheral IV line. However, for repeat doses of HTS with concentrations ≥5%, the use of a central IV line is recommended, if available. NS = Normal Saline; HTS = Hypertonic Saline.

Hypertonic Saline Preparations			
**Concentration**	3%	5%	7.5%
**Preparation**	100 mL of NaCl at a concentration of 2 mEq/mL + 400 mL of NS	200 mL of NaCl at a concentration of 2 mEq/mL + 300 mL of NS	300 mL of NaCl at a concentration of 2 mEq/mL + 200 mL of NS
**Dose**	4-5 mL/kg	3-4 mL/kg	2 mL/kg

**Table 9:** **Criteria for Initiating Early Anticonvulsant Prophylaxis.** Prophylaxis should be maintained for 7 days.

**Criteria for Initiating Early Anticonvulsant Prophylaxis**	● Severe TBI ● Moderate or mild TBI that meets any of the following conditions: ○ Depressed skull fracture ○ Penetrating TBI ○ Intracranial bleeding ○ Post-traumatic seizure
**Recommended Medications for Anticonvulsant Prophylaxis**	**Levetiracetam:** 1 g every 12 h. **Phenytoin:** Initial loading dose of 15 mg/kg divided into 3 doses: (half at hour 0, a quarter at hour 8, and the last quarter at hour 16). The maintenance dose is 5 mg/kg/day divided into 3 daily doses.

**Table 10:** Denver Criteria for blunt cerebrovascular injury.

Denver Criteria for Blunt Cerebrovascular Injury	
Signs and Symptoms	Risk Factors
Arterial hemorrhage	Midface Fractures (Le Fort II or III)
Cervical bruit	Basilar Skull Fracture with carotid canal involvement
Expanding neck hematoma	Diffuse axonal injury with GCS <6
Focal neurologic deficit	Cervical spine fracture
Neurological exam findings inconsistent with CT findings	Hanging with anoxic brain injury
Stroke on neuroimaging	Seat belt abrasion or other soft tissue injury of the anterior neck resulting in significant swelling or altered mental status
**Interpretation:** If a trauma patient meets any of these criteria, the possibility of blunt cerebrovascular injury should be evaluated with CT Angiography.

**Table 11:** **Suggested Indications for Neurological Surgery.** ∗The choice between intraparenchymal and intraventricular catheters will be guided by surgical criteria, including ventricular patency, accessibility, and the need for CSF drainage as a therapeutic measure. mGCS = Motor Glasgow Coma Scale; MLS = Midline Shift; ZI = Zumkeller Index (ratio of hematoma volume to estimated tolerable hematoma volume based on CT measurements) (Zumkeller et al., 1996); TBI = Traumatic Brain Injury; ONSD = Optic Nerve Sheath Diameter; NPi = Neurological Pupil Index; MCV = Maximum Constriction Velocity; MCA = Middle Cerebral Artery; PI = Pulsatility Index; EDV = End-Diastolic Velocity.

Craniectomy	Craniotomy	Cisternostomy	External Ventricular Drain	Intraparenchymal ICP ± PbtO_2_ monitor
Large intracranial hematoma + Zumkeller Index >3	Large intracranial hematoma with Zi < 3	Large peri-mesencephalic tSAH + Persistent impaired intracranial compliance despite optimal medical therapy	GCS ≤8 + Abnormal CT scan∗	GCS ≤8 + Abnormal CT scan∗
Significant cerebral edema on CT imaging			GCS ≤8 + Two or more of the following are present∗: Age >40 years Abnormal motor posturing SBP <90 mm Hg	GCS ≤8 + Two or more of the following are present∗: Age >40 years Abnormal motor posturing SBP <90 mm Hg
Persistent intracranial compliance impairment despite optimal medical therapy, hematoma drainage and/or EVD	Intracranial hematoma of any size without significant cerebral edema on CT + Persistent impaired intracranial compliance despite optimal medical therapy		Patients with moderate to severe TBI in whom serial neurological examinations are not feasible, such as those requiring sedation or mechanical ventilation∗	Patients with moderate to severe TBI in whom serial neurological examinations are not feasible, such as those requiring sedation or mechanical ventilation∗
Posterior fossa hematoma >10 mL and hydrocephalus			Persistent intracranial compliance impairment with normal/near-normal CT despite optimal medical therapy and/or hematoma drainage	
Persistent PbtO_2_ < 20 mmHg not attributable to systemic causes				
**Other indications:** ● Wounds with brain exposure (open brain injury) ● Complex scalp wound associated with underlying skull fracture				
**Interpretation:** ● **Large intracranial hematoma:** ○ Epidural hematoma >30 mL ○ Intracerebral hematoma or contusion >50 mL ○ Subdural hematoma >10 mm ● **Significant cerebral edema:** ○ MLS ≥5 mm ○ Basal cistern effacement (grade III edema) ● **Intracranial compliance impairment:** abnormal findings in any combination of ≥2 non-invasive monitoring systems or abnormal ICP waveform				

**Note:** emergency surgery must be performed **immediately** in cases of non-responsive unilateral mydriasis.

**Table 12:** **Neuroprotective Goals.** Hb = Hemoglobin; Na = Sodium; P_50_ = Oxygen-Hemoglobin Dissociation Curve; MAP = Mean Arterial Pressure; SBP = Systolic Blood Pressure; CPP = Cerebral Perfusion Pressure; PbtO2 = Partial pressure of brain tissue oxygen; PEEP = Positive End-Expiratory Pressure; PT = Prothrombin Time; aPTT = Activated Partial Thromboplastin Time; PaO_2_ = Arterial Oxygen Partial Pressure; RASS = Richmond Agitation-Sedation Scale; RR = Respiratory Rate; SpO_2_ = Peripheral Oxygen Saturation; PaCO_2_ =Arterial Carbon Dioxide Partial Pressure; TV = Tidal Volume.

● Maintain head elevation at 30–45° with the body axis in neutral alignment
● RASS -5
● Maintain temperature between 36 and 37 °C
● Hb target ≥9 g/dL
● Maintain Na 135-155 mEq/dL
● Keep normal levels of other electrolytes
● Keep lactate levels less than 2 mmol/L
● Blood pH 7.35-7.45
● P_50_ target 26-28
● MAP >80 mmHg and SBP >100 mmHg
● CPP between 60 and 70 mmHg and varies according to autoregulatory status
● Maintain blood glucose between 110 and 170 mg/dL
● Keep normal levels of coagulation tests: INR less than 1.5, platelets more than 100,000/UL and fibrinogen >150 mg
● PaO_2_ 80-110 mmHg
● SpO_2_ > 94%
● PaCO_2_ 35-45 mmHg
● ICP should be maintained at < 22 mmHg, or further reduced if abnormal ICP waveform patterns are observed
● Maintain PbtO_2_ >20 mmHg whenever monitoring is available
● Maintain venous jugular oxygen saturation (SjO2) > 55%
● Neuroprotective Ventilatory Parameters
○ RR: 10-20 bpm
○ PaCO_2_: 35 - 45 mmHg
○ TV: 5 - 7 ml/kg
○ PEEP: 5 cmH_2_O
○ PaO_2_ > 60 mmHg
○ SpO_2_ > 94%

Management should begin with an effective handoff from the prehospital team, ensuring completion of the TBI triage checklist if the patient is stable. Simultaneously, telemedicine support should be activated when available.

A proactive evaluation for neurological warning signs, CT scan criteria, and the Denver criteria for suspected intracranial vascular injury is strongly recommended (Table 2, Table 10) (Brommeland et al., 2018). The use of noninvasive neuromonitoring tools and serum biomarkers is also advised to help identify patients who require transfer to higher-complexity facilities. Important note: The diagnostic thresholds of serum biomarkers (GFAP and UCH-L1) are time-dependent and must be interpreted in relation to the time elapsed since injury, not as absolute values independent of the time of blood draw. These thresholds (GFAP >30 pg/mL and UCH-L1 >360 pg/mL, corresponding to Abbott TBI Plasma Test cutoffs) are validated exclusively within 12 h of injury in patients with GCS 13–15. Beyond this window, particularly for UCH-L1 (half-life ∼6–7 h), a negative result cannot reliably exclude intracranial injury and should not replace clinical judgment or CT imaging when indicated (Welch et al., 2025).

##### 3.1.2.3 Questions 4 and 5

● What is the best up-to-date protocol for managing a patient with a head injury in a medium-complexity emergency department, where intermediate equipment (e.g., Computed Tomography) is available and only some staff are trained in advanced interventions?
● What is the best up-to-date protocol for managing a patient with a head injury in a high-complexity emergency department, where advanced equipment is available and all staff are fully trained in advanced interventions?

##### 3.1.2.4 Recommendations

**Fig. 6:**
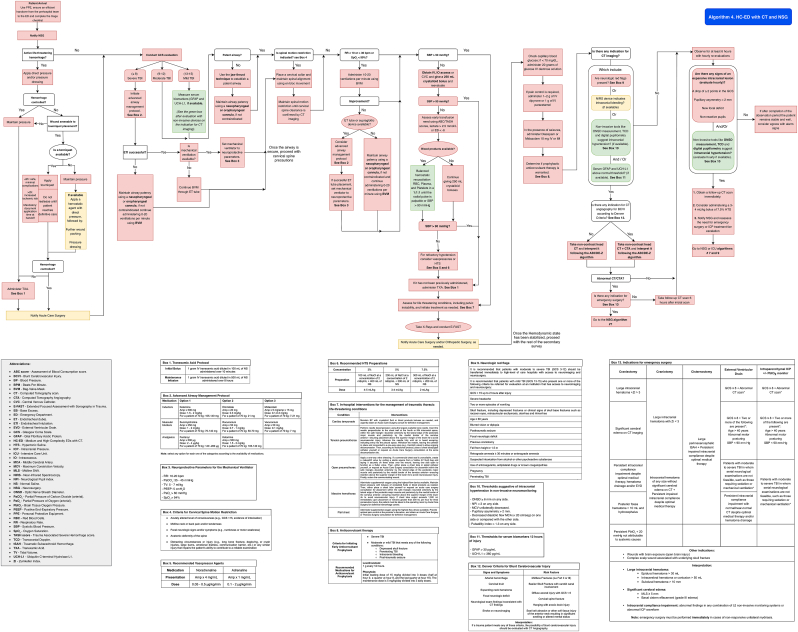
Management algorithm for patients with traumatic brain injury in medium and high-complexity EDs with CT (algorithm 4).

Patients with TBI presenting to medium- and high-complexity emergency departments should be managed according to Fig. 6 (Algorithm 4).

**Fig. 7:**
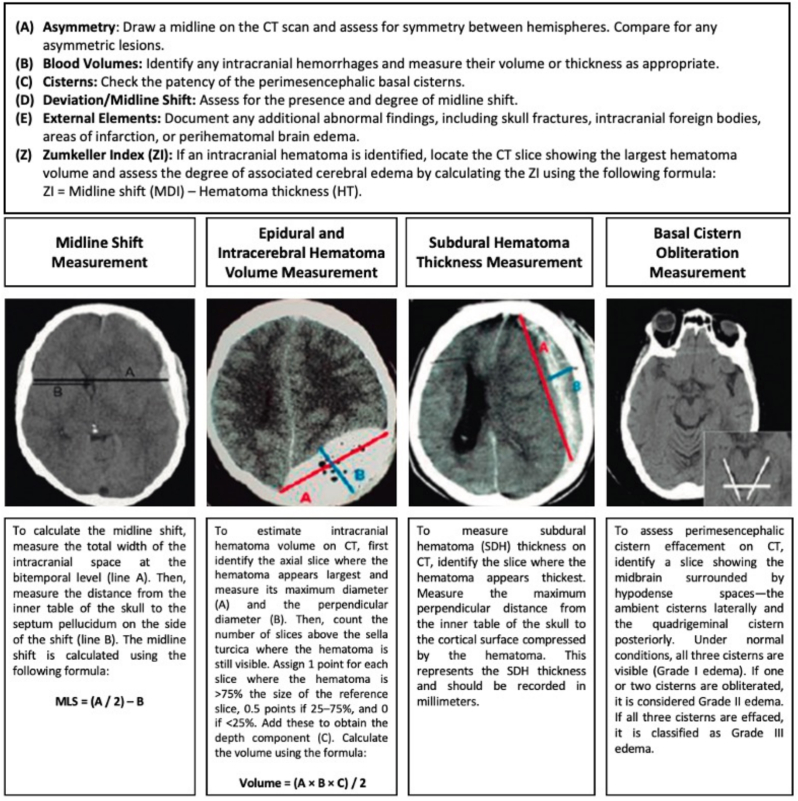
ABCDE-Z method for CT scan interpretation.

The interpretation of head CT scans by emergency personnel in TBI patients should be standardized using the ABCDE method to ensure a systematic and comprehensive evaluation, as seen in Fig. 7.

#### 3.1.3 Neurological surgery

Indications for emergency surgery are shown in Table 11.

##### 3.1.3.1 Question 6

● What is the best up-to-date protocol for managing a patient with a head injury requiring urgent surgery in a facility without neurosurgical services, but where general surgery and CT imaging are available?

##### 3.1.3.2 Recommendations

In many low-income or rural settings, hospitals lack neurosurgical services. In such cases, it is recommended to activate a telemedicine protocol, if available, to obtain remote specialist guidance while simultaneously initiating urgent referral procedures to a facility equipped with neurosurgical capabilities.

If telemedicine is not available, management should focus on achieving baseline neuroprotection goals and guiding therapy based on clinical examination findings without delaying urgent referral procedures to a facility equipped with neurosurgical capabilities.

Exploratory trepanation by non-neurosurgeons is not recommended. It should only be considered under telemedicine guidance in situations where timely transfer is not feasible and if permitted by national legislation.

When the referral center is located more than 150 km away, air transport should be considered to reduce delays in definitive care.

**Fig. 8:**
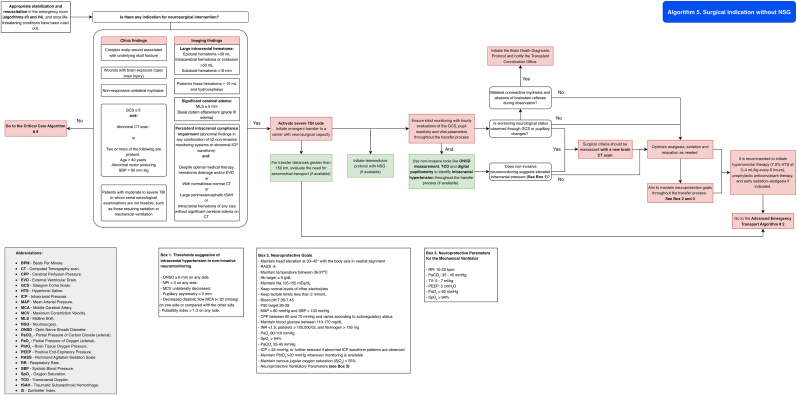
Management algorithm for patients with traumatic brain injury requiring surgery at centers without neurosurgical capacity (algorithm 5).

It is recommended to follow the algorithm shown in Fig. 8 (Algorithm 5).

##### 3.1.3.3 Question 7

● What is the best up-to-date protocol for managing a patient with a head injury requiring urgent surgery in a facility with neurosurgical capabilities, but without access to an ICU?

##### 3.1.3.4 Recommendations

Patients with neurosurgical indications (Table 11) who are treated at centers with neurosurgical capabilities but without access to an ICU should be referred to a facility that provides both services.

If the estimated time from the moment of injury to arrival at the referral center exceeds 4 h, it is recommended that surgical intervention be performed at the current facility, followed by postoperative referral.

Postoperatively, patients should remain sedated and monitored in the recovery room or operating room while arrangements are made for transfer to a center with ICU capabilities.

**Fig. 9:**
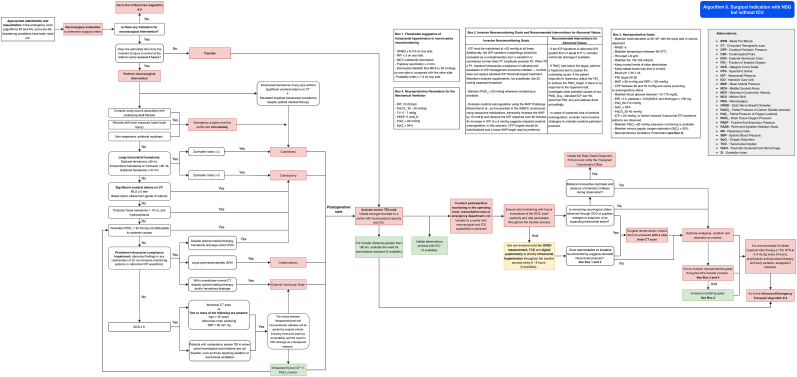
Management algorithm for traumatic brain injury patients requiring surgery at centers with neurosurgical but without intensive care capacity (algorithm 6).

See the algorithm shown in Fig. 9 (Algorithm 6) and Table 12, Table 13.

**Table 13:** Invasive neuromonitoring goals and recommended interventions for abnormal values.

Invasive Neuromonitoring Goals	Recommended Interventions for Abnormal Values
● ICP must be maintained at <22 mmHg. Additionally, the ICP waveform morphology should be evaluated as a complementary tool: a waveform is considered normal when P1 amplitude exceeds P2. When P2 ≥ P1, impaired intracranial compliance is indicated and escalation of ICP management should be initiated (analyzed in conjunction with the absolute ICP threshold). Waveform analysis supplements the 22-mmHg treatment threshold (Chesnut et al., 2020a; Hawryluk et al., 2019; Brasil et al., 2021). ● Maintain PbtO_2_ > 20 mmHg whenever monitoring is available. ● Evaluate cerebral autoregulation using the MAP Challenge (Rosenthal et al., as incorporated in the SIBICC consensus) (Chesnut et al., 2020a; Rosenthal et al., 2011): using vasoactive medications, transiently increasing the MAP by 10 mmHg and observe the ICP response over 20 min. An increase in ICP of ≥2 mmHg suggests impaired cerebral autoregulation; in this scenario, CPP targets should be individualized, and a lower MAP target may be preferred.	● If the ICP waveform is abnormal (P2 greater than or equal to P1), consider ventricular drainage if available. ● If PbtO_2_ falls below the target, perform a hyperoxia test to assess the underlying cause. If the patient responds to hyperoxia, adjust the FiO_2_ to achieve the PbtO_2_ target. If there is no response to the hyperoxia test, investigate other potential causes of low PbtO_2_ (e.g., elevated ICP, low Hb, abnormal P_50_, etc) and address them accordingly. ● In cases of sustained loss of cerebral autoregulation, consider more invasive strategies to maintain cerebral perfusion pressure.

##### 3.1.3.5 Question 8

● What is the best up-to-date protocol for managing a patient with a head injury requiring urgent surgery in a fully equipped facility with immediate access to neurosurgery, MRI, and ICU care?

##### 3.1.3.6 Recommendations

**Fig. 10:**
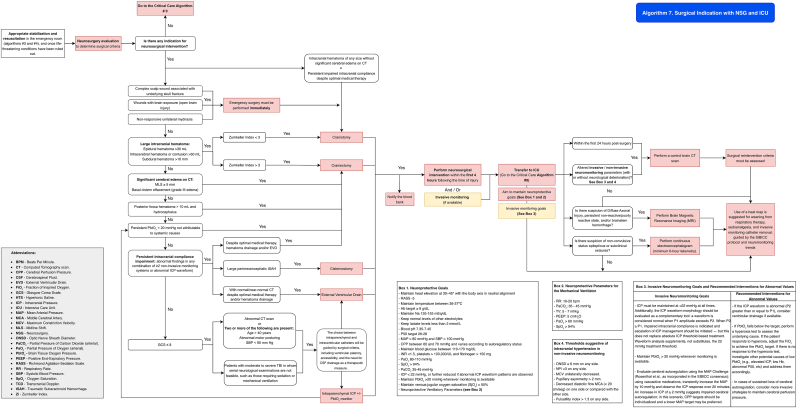
Management algorithm for traumatic brain injury patients requiring surgery at centers with neurosurgical and intensive care capacity (algorithm 7).

It is recommended that patients with a neurosurgical indication undergo surgical intervention immediately after hemodynamic stabilization. See Fig. 10 (Algorithm 7) and Table 12, Table 13

#### 3.1.4 Intensive care

##### 3.1.4.1 Questions 9 and 10

● What is the best up-to-date protocol for managing a head injury patient in a postoperative recovery area equipped with only basic monitoring and staffed by personnel without ICU-specific training?
● What is the best up-to-date protocol for managing a head injury patient in an intermediate care unit, where monitoring equipment is moderately advanced and only some staff have ICU-level training?

##### 3.1.4.2 Recommendations

**Fig. 11:**
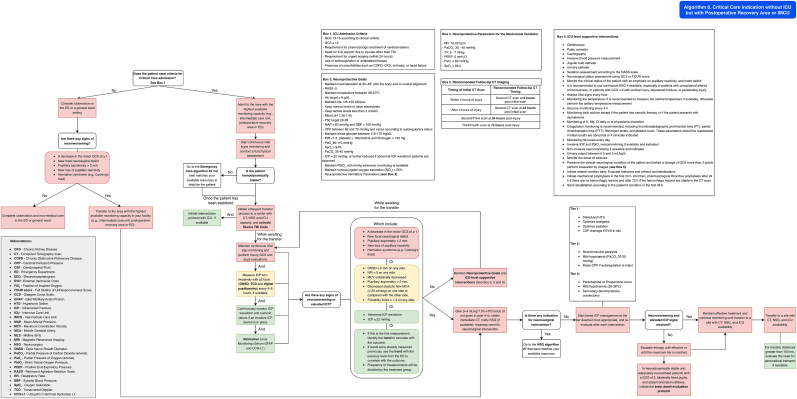
Management algorithm for traumatic brain injury patients requiring ICU-level care at centers without ICU but with postoperative recovery area capacity or intermediate care capacity (algorithm 8).

Patients with indications for ICU admission (Table 14) who are in centers without ICU availability but have access to a postoperative recovery area or an Intermediate Care Unit (IMCU) should be managed in that setting while awaiting transfer. During this period, ICU-level supportive interventions (Table 15) should be initiated, and efforts should be made to achieve hemodynamic, ventilatory, metabolic, and neuroprotection goals (Table 9, Table 12, Table 13), following the guidance of Fig. 11 (Algorithm 8). The primary objective is to prevent secondary brain injury and stabilize the patient's condition. Whenever possible, management should be supported through telemedicine consultation.

**Table 14:** ICU Admission Criteria. CKD = chronic kidney disease; COPD = Chronic Obstructive Pulmonary Disease; TBI = Traumatic Brain Injury.● GCS 13-15 according to clinical criteria.

● GCS ≤12
● Requirement for pharmacologic treatment of cerebral edema
● Need for ICU support due to injuries other than TBI
● Requirement for urgent surgery (within 24 h)
● Use of anticoagulation or antiplatelet therapy
● Presence of comorbidities such as COPD, CKD, cirrhosis, or heart failure

**Table 15:** **ICU-level supportive interventions.** Abbreviations: RASS = Richmond Agitation-Sedation Scale; GCS = Glasgow Coma Scale; FOUR = Full Outline of Un Responsiveness score; EEG = Electroencephalogram; ICP = Intracranial Pressure; PbtO_2_ = Brain Tissue Oxygen Pressure; PT = Prothrombin Time; PTT = Partial Thromboplastin Time; Hb = Hemoglobin; CT = Computed Tomography; K = Potassium; Mg = Magnesium; Cl = Chloride.

Cardioscope
Pulse oximeter
Capnography
Invasive blood pressure measurement
Jugular bulb catheter
Urinary catheter
Sedation assessment according to the RASS scale
Neurological status assessment using GCS or FOUR score
Monitor the clinical status of the patient with an emphasis on pupillary reactivity, and motor deficit
It is recommended to use continuous EEG if available, especially in patients with unexplained altered consciousness, or patients with GCS ≤8 with cortical injury, depressed fracture, or penetrating injury
Assess vital signs every hour
Monitoring the temperature: It is recommended to measure the central temperature if available, otherwise perform the axillary temperature measurement
Glucose monitoring every 4 h
Monitoring daily sodium except if the patient has osmotic therapy or if the patient does not have disnatremias
Monitoring of K, Mg, Cl daily or at physician's discretion
Coagulation monitoring is recommended, including Thromboelastography, prothrombin time (PT), partial thromboplastin time (PTT), fibrinogen levels, and platelet count. These parameters should be reassessed if initial results are abnormal or if clinically indicated
Monitoring Hb levels every day
Invasive ICP and PbtO_2_ neuromonitoring if available and indicated
Non-invasive neuromonitoring if available and indicated
Urinary output between 0.5 and 3 mL/kg/h
Monitor the onset of seizures, and if it has EEG indications
Preserve the clinical neurological condition of the patient and before a change of GCS more than 2 points perform evaluation by images
Initiate enteral nutrition early. Evaluate tolerance and without contraindications
Initiate mechanical prophylaxis in the first 24 h. And then, pharmacological thrombus prophylaxis after 24 h if there are no hemorrhagic lesions and after 72 h if the hemorrhagic lesions are stable in the CT scan
Evaluation and rehabilitation, according to the patient's condition in the first 48 h

##### 3.1.4.3 Question 11

● What is the best up-to-date protocol for managing a head injury patient in a general intensive care unit, where advanced equipment is available and all staff are fully trained in ICU-level care?

##### 3.1.4.4 Recommendations

**Fig. 12:**
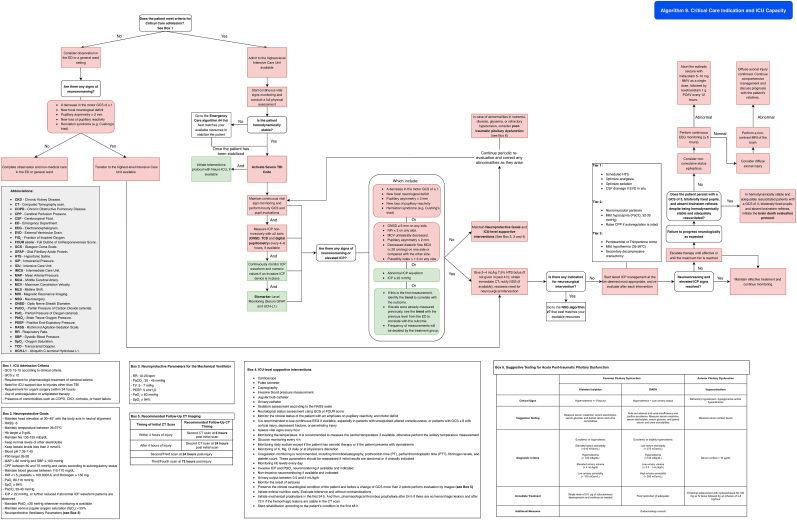
Management algorithm for traumatic brain injury patients requiring ICU-level care in high-complexity centers (algorithm 9).

Patients in centers with ICU availability should be managed according to Fig. 12 (Algorithm 9).

In facilities equipped with invasive intracranial pressure (ICP) and brain tissue oxygen pressure (PbtO_2_) monitoring, it is recommended to assess whether these modalities are indicated for the individual patient (Table 16). When used, they should serve as tools to guide goal-directed therapy (Table 13).

**Table 16:** **Indications for Invasive Neuromonitoring in TBI Patients.** The choice between intraparenchymal and intraventricular catheters will be guided by surgical criteria, including ventricular patency, accessibility, and the need for CSF drainage as a therapeutic measure.

● GCS ≤8 + Abnormal CT scan
● GCS ≤8 + Two or more of the following are present:
○ Age >40 years
○ Abnormal motor posturing
○ MAP <90 mm Hg
● Patients with moderate to severe TBI in whom serial neurological examinations are not feasible, such as those requiring sedation or mechanical ventilation.

## 4 Discussion

Current evidence-based guidelines for the treatment of TBI are primarily based on studies conducted in HICs, where clinical resources are readily available (Carney et al., 2016; Le Roux et al., 2014a; Claassen et al., 2013; Andrews et al., 2008; Potapov et al., 2015; Potapov et al., 2016a; Potapov et al., 2016b; Le Roux et al., 2014b; Stocchetti et al., 2014; The Participants in the International Multidisciplinary Consensus Conference on Multimodality Monitoring et al., 2014; Chesnut et al., 2015; Chesnut et al., 2020b). However, over 80% of the global population lives in LMICs, where healthcare resources are often limited and unpredictable. A global survey of 803 neurosurgeons confirmed that adherence to current TBI guidelines differs significantly between LMICs and high-income countries, with the authors concluding that guidelines need to better reflect the realities of different resource environments (Saraceno et al., 2021). Few guidelines specifically address the unique challenges of managing TBI in these settings, and even those that attempt to do so frequently fall short, as their methodologies, rooted in evidence-based frameworks, may not fully reflect the realities of clinical care in resource-constrained environments. In LMICs, essential resources such as medications, equipment, and trained personnel may not always be reliably accessible when needed.

The BOOTStraP initiative seeks to bridge these gaps using a mixed-methods approach that combines the best available evidence with expert consensus, resulting in context-specific recommendations for TBI management across varying resource settings. BOOTStraP is structured as a two-dimensional algorithm, with one dimension representing the phases of care - prehospital, ED, surgery, and ICU, and the second reflecting the level of resource availability, which can be dynamic and unpredictable in LMICs. Even well-resourced centers may face sudden shortages or limitations in specific areas, and some systems may be well-equipped during one phase of care (e.g., ED) but under-resourced in another (e.g., emergency transport or ICU). While this two-dimensional model, shown in Fig. 1, is a simplification, it offers a practical framework for guiding treatment strategies within the BOOTStraP model.

A key strength of this project is the involvement of experts from a wide range of specialties and healthcare settings, which enhances the relevance of the guidelines by integrating multiple perspectives. This multidisciplinary collaboration ensures that the recommendations are closely aligned with real-world clinical scenarios. Additionally, the use of color coding in the algorithms and supplementary materials improves usability, allowing practitioners to adapt the guidance to their specific resource contexts. Building on insights from the previous version of the consensus, this iteration incorporates updates reflecting recent advances in neurotraumatology and addresses prior limitations, such as the overly complex structure of the treatment algorithms. As was previously highlighted, these algorithms are designed as clinical decision-support tools and each recommendation must be interpreted in the context of the individual patient's clinical status and the evolving clinical situation. The algorithms should be used in conjunction with (not as a substitute for) individualized patient assessment.

Surgical decision-making in the most critical patients with severe TBI (GCS 3–5 post-resuscitation) remains among the most challenging scenarios in neurotrauma, particularly in resource-limited settings where advanced monitoring is unavailable. A recent systematic review highlighted the persistent uncertainty surrounding neurosurgical intervention in this subgroup (Gkantsinikoudis et al., 2024). The BOOTStraP surgical indications table (Table 11) addresses this by providing explicit clinical and imaging criteria, including non-invasive monitoring thresholds, stratified by resource level, to guide surgical decision-making in precisely these high-stakes scenarios.

The BOOTStraP framework has demonstrated sufficient utility to inspire parallel adaptations for other neurotrauma contexts. Using the same resource-stratification and consensus methodology, the BOOTStrap-SCI initiative has recently produced stratified protocols for the management of spinal trauma and spinal cord injury across prehospital, emergency, surgical, and intensive care phases (Marchesini et al., 2025a, 2025b). This parallel development reinforces the validity of the resource-stratified approach as a generalized model for addressing clinical decision-making gaps across the spectrum of traumatic neurological injury. Similarly, recent consensus work on TBI management in older adults (a population increasingly affected given global demographic trends) highlights both the methodological utility of Delphi-based consensus processes and the existence of important subgroup-specific knowledge gaps not yet addressed in resource-stratified protocols (Lagares et al., 2025). The management of older TBI patients in low-resource settings remains an area for future development of the BOOTStraP framework.

Limitations of the project include the exclusion of pediatric patients (under 15 years of age), the non-inclusion of experimental therapies or tools, and the absence of direct recommendations for primary prevention and rehabilitation, as these aspects fall outside the defined scope of this consensus. The open, in-person voting methodology inherent to the Nominal Group Technique carries a recognized risk of social desirability bias, which is acknowledged as a methodological limitation. Furthermore, the Scandinavian Neurotrauma Committee guidelines — a validated clinical decision tool for mild TBI — illustrate that even well-established protocols may face significant adherence challenges in real-world settings, in part due to biomarker availability constraints (Josefsson et al., 2025). This reinforces the importance of feasibility assessment and local adaptation in the implementation of BOOTStraP.

Looking ahead, we aim to continue disseminating the BOOTStraP framework globally, adapting optimal TBI care to diverse resource settings with support from international collaborators such as the World Federation of Neurosurgical Societies (WFNS) and the Global Health Research Group on Acquired Brain and Spine Injuries (NIHR-UK). A pilot study evaluating the feasibility and initial impact of implementing the first edition of BOOTStraP across 3 centers in different cities in Colombia was conducted between 2021 and 2022, and its results are expected to be published in 2026. Important implementation challenges identified in the pilot included variability in telemedicine infrastructure, inconsistent availability of key medications, and the need for structured training programs to ensure correct color-coded algorithm interpretation. Notably, the algorithms are intended as decision-support tools requiring local adaptation and clinical judgment; they are not designed to replace individualized patient assessment. Institutions adopting these protocols should prospectively evaluate adherence and outcomes to generate local validation data. The algorithms are not a substitute for institutional protocol development and should be adapted to local regulatory, resource, and training contexts. Building on this foundation, we plan to expand to a larger multicenter implementation study. The current exercise of BOOTStrap has been followed in Peru, Ecuador, Paraguay and Brazil, and the specific exercises are in process of being published and the complete final version in local language has been submitted to the local governments for adoption a nation level on each country.

To support the implementation of this protocol in diverse healthcare settings, an Implementation Checklist is provided as Supplementary Material S4. The checklist covers eight domains: staff training on color-coded algorithm interpretation, medication availability audit, telemedicine connectivity, non-invasive neuromonitoring equipment, serum biomarker availability, inter-facility transfers protocols, outcome tracking, and local adaptation documentation.

## 5 Conclusion

Current evidence-based guidelines for the treatment of TBI often contain significant limitations, particularly in areas where evidence is scarce or lacking. The transferability of these guidelines to real-world clinical practice is frequently compromised by a disconnection between the recommendations and the actual constraints of healthcare systems, particularly where training and resources are limited. To address these gaps, the development of expert consensus-based recommendations becomes a valuable strategy, even in settings where resources or specialized training are scarce.

A practical method to bridge these gaps is through the stratification of interventions based on the availability of resources at different stages of care. This approach facilitates the development of more context-appropriate treatment strategies for TBI in real-life scenarios. In this guideline, we present 9 management algorithms based on expert consensus, designed to support TBI protocols across prehospital care, emergency departments, neurosurgery, and intensive care. These algorithms offer flexible, resource-sensitive treatment options, ensuring that care can be appropriately adapted to the available resources, regardless of the level of care or setting.

## Declaration of competing interest

The authors declare that they have no known competing financial interests or personal relationships that could have appeared to influence the work reported in this paper.
