# Supplementary material for: Second edition of the recommendations from the Colombian consensus committee for the management of traumatic brain injury in the prehospital setting, emergency department, surgery, and intensive care (Beyond one option for treatment of traumatic brain injury: A stratified protocol [BOOTStraP])
Source: Brain Spine. 2026 Apr 12;6:106046. doi: 10.1016/j.bas.2026.106046 (PMC13101607; doi:10.1016/j.bas.2026.106046)
Supplement: Multimedia component 1 [file mmc1.docx]

**Supplementary material S1: Voting Process and Consensus Results**

1. **Overview of the Consensus Process**

This supplementary material documents the structure, methodology, and outcomes of the voting process used to develop the recommendations of the second edition of BOOTStraP (Beyond One Option for Treatment of Traumatic Brain Injury: A Stratified Protocol).

The consensus process was conducted in August 2023 in Cali, Colombia, following the principles of the Delphi method and the Nominal Group Technique (NGT), consistent with the methodology used in the first BOOTStraP edition (Rubiano et al., J Neurosci Rural Pract 2020).

1. **Participant Composition**

| **Domain** | **Experts (n)** | **Moderator** | **Algorithm(s)** |
| --- | --- | --- | --- |
| Prehospital Care | 3 | **Yes** | Alg. 1–2 |
| Emergency Medicine | 4 | **Yes** | Alg. 3–4 |
| Neurological Surgery | 5 | **Yes** | Alg. 5–7 |
| Intensive Care | 5 | **Yes** | Alg. 8–9 |
| **Methodological facilitators** | 2 | N/A | All |
| **TOTAL** | **17** | **4** | **9** |

1. **Voting Procedure**
   1. **Meeting Structure:**

The consensus conference took place over two days: Day 1 – Subgroup sessions: Each of the four domain subgroups worked independently to draft preliminary recommendations for their assigned questions, using preparatory materials distributed one month in advance (CPGs, systematic reviews, and key publications relevant to 2019–2023 advances in neurotraumatology).

Day 2 – Plenary session: Representatives of each subgroup presented their draft recommendations to all 17 participants. Each recommendation was discussed collectively and then submitted to a final vote.

- 1. **Voting Method**

Voting was conducted in-person by a show of hands in accordance with the Nominal Group Technique (NGT). This open, face-to-face voting format is a defining methodological feature of the NGT and is designed to:

● Allow immediate discussion of disagreements between voting rounds;

● Enable the group to reach progressive convergence through iterative deliberation;

● Facilitate real-time facilitation by methodological team members.

Each INDIVIDUAL recommendation item was voted on separately. Composite algorithms were assembled only after each constituent item independently achieved the required agreement threshold. This ensured that no single item was 'carried' by the overall approval of an algorithm.

Methodological facilitators (n=2, without domain expertise) recorded all votes using paper tallies and announced results immediately after each vote.

- 1. **Agreement thresholds**

| **Voting Stage** | **Required Agreement** | **Consequence if not met** |
| --- | --- | --- |
| Subgroup internal vote (Day 1) | **≥70% of subgroup members** | Item re-discussed and re-voted; modified or excluded |
| Full plenary vote (Day 2) | **≥90% of all 17 participants** | Item returned to subgroup for revision; re-presented in next round |

- 1. **Limitation Acknowledgment**

Open show-of-hands voting is inherently non-anonymous and may carry a risk of social desirability bias or anchoring to early speakers' positions. This is a recognized limitation of the Nominal Group Technique, acknowledged in the manuscript's Limitations section. Mitigation strategies employed included: (i) structured round-robin initial statements before discussion; (ii) written position statements submitted before plenary by each subgroup; (iii) facilitated moderator neutrality; and (iv) requirement for iterative rounds when initial agreement was not reached.

1. **Voting results by question and recommendation item**

All items listed below achieved the required agreement thresholds at both subgroup (≥70%) and plenary (≥90%) levels. No recommendation item was excluded due to failure to reach consensus.

**PHASE 1 – PREHOSPITAL CARE**

*Prehospital care subgroup (n = 3 experts + 1 moderator)*

**Q1 —** *What is the best up-to-date protocol for managing a patient with a head injury during basic emergency transport (BET), where only essential equipment is available and personnel lack training in advanced interventions?*

Algorithm produced: Algorithm 1 – Figure 2

| **Recommendation item voted on** | **Subgroup (≥70%)** | **Plenary (≥90%)** | **Consensus** |
| --- | --- | --- | --- |
| Initial approach with PPE, safety perimeter, and risk factor identification | **100%** | **100%** | **✓ CONSENSUS ACHIEVED** |
| Initiation of telemedicine protocol with ED if available | **100%** | **94% (16/17)** | **✓ CONSENSUS ACHIEVED** |
| Assessment of massive hemorrhage and initiation of hemorrhage control protocol | **75%** | **100%** | **✓ CONSENSUS ACHIEVED** |
| Airway patency assessment in absence of hemorrhage | **100%** | **100%** | **✓ CONSENSUS ACHIEVED** |
| Consciousness level assessment (AVPU/GCS); jaw-thrust and supraglottic devices for GCS ≤8 | **80%** | **100%** | **✓ CONSENSUS ACHIEVED** |
| SpO₂ assessment; supplemental oxygen if SpO₂ <94% | **100%** | **100%** | **✓ CONSENSUS ACHIEVED** |
| Spinal motion restriction (SMR) criteria assessment and implementation | **100%** | **100%** | **✓ CONSENSUS ACHIEVED** |
| Ventilation and thoracic injury assessment; corrective measures if identified | **80%** | **100%** | **✓ CONSENSUS ACHIEVED** |
| Ventilatory failure assessment; BVM ventilation targeting RR 10–20 and SpO₂ >94% | **100%** | **100%** | **✓ CONSENSUS ACHIEVED** |
| Radial pulse/SBP assessment; IV/IO access and fluid therapy for SBP <90 mmHg | **80%** | **94% (16/17)** | **✓ CONSENSUS ACHIEVED** |
| Capillary glucose check; dextrose bolus for hypoglycemia <70 mg/dL | **80%** | **100%** | **✓ CONSENSUS ACHIEVED** |
| Complete neurological assessment (pupils, motor); urgent transfer criteria | **100%** | **100%** | **✓ CONSENSUS ACHIEVED** |
| NIRS for intracranial hematoma detection if available; urgent transfer if positive | **80%** | **100%** | **✓ CONSENSUS ACHIEVED** |
| Seizure monitoring and management with midazolam/diazepam | **80%** | **100%** | **✓ CONSENSUS ACHIEVED** |
| Pain assessment and analgesia (dipyrone/paracetamol/acetaminophen) | **80%** | **100%** | **✓ CONSENSUS ACHIEVED** |
| Patient exposure, temperature control, secondary survey | **100%** | **100%** | **✓ CONSENSUS ACHIEVED** |
| Ground transfer; priority to advanced center with neurosurgery/CT/ICU | **100%** | **100%** | **✓ CONSENSUS ACHIEVED** |
| Aeromedical evacuation if distance >150 km to advanced center | **75%** | **100%** | **✓ CONSENSUS ACHIEVED** |
| Documentation of care provided in physical or digital format | **80%** | **100%** | **✓ CONSENSUS ACHIEVED** |

**Q2 —** *What is the best up-to-date protocol for managing a patient with a head injury during advanced emergency transport (AET), where personnel are fully trained and equipped to perform advanced interventions?*

Algorithm produced: Algorithm 2 – Figure 4

| **Recommendation item voted on** | **Subgroup (100%)** | **Plenary (100%)** | **Consensus** |
| --- | --- | --- | --- |
| Initial approach with PPE, safety perimeter, and risk factor identification | **100%** | **R1: 82% → R2: 94%** | **✓ CONSENSUS ACHIEVED** |
| Initiation of telemedicine protocol with ED if available | **100%** | **94% (16/17)** | **✓ CONSENSUS ACHIEVED** |
| Assessment of massive hemorrhage and initiation of hemorrhage control protocol | **100%** | **94% (16/17)** | **✓ CONSENSUS ACHIEVED** |
| Airway patency assessment in absence of hemorrhage | **100%** | **100%** | **✓ CONSENSUS ACHIEVED** |
| GCS assessment; jaw-thrust, supraglottic devices, or orotracheal intubation (RSI) for GCS ≤8 | **75%** | **100%** | **✓ CONSENSUS ACHIEVED** |
| SpO₂ assessment; supplemental oxygen if SpO₂ <94% | **100%** | **100%** | **✓ CONSENSUS ACHIEVED** |
| SMR criteria assessment and implementation | **75%** | **94% (16/17)** | **✓ CONSENSUS ACHIEVED** |
| Ventilation and thoracic injury assessment; corrective measures if identified | **100%** | **94% (16/17)** | **✓ CONSENSUS ACHIEVED** |
| Ventilatory failure assessment; BVM or RSI targeting RR 10–20 and SpO₂ >94% | **75%** | **100%** | **✓ CONSENSUS ACHIEVED** |
| SBP measurement; IV/IO access and fluid therapy for SBP <90 mmHg | **100%** | **100%** | **✓ CONSENSUS ACHIEVED** |
| TXA 1g bolus + 1g infusion for mild-moderate TBI within 3 hours; lyophilized plasma/3% HTS if available | **100%** | **100%** | **✓ CONSENSUS ACHIEVED** |
| Capillary glucose check; dextrose bolus for hypoglycemia; target 110–180 mg/dL | **100%** | **100%** | **✓ CONSENSUS ACHIEVED** |
| Complete neurological assessment; seizure management with diazepam/midazolam | **80%** | **R1: 76% → R2: 100%** | **✓ CONSENSUS ACHIEVED** |
| Motor deficit assessment; urgent transfer criteria (GCS ≤8, hemiplegia, anisocoria >2mm) | **80%** | **100%** | **✓ CONSENSUS ACHIEVED** |
| NIRS for intracranial hematoma detection if available | **80%** | **100%** | **✓ CONSENSUS ACHIEVED** |
| Non-invasive neuromonitoring: ONSD ≥6mm, NPi <3, anisocoria >2mm, decreased MCV → 3% HTS and urgent transfer | **100%** | **94% (16/17)** | **✓ CONSENSUS ACHIEVED** |
| Pain assessment and analgesia | **100%** | **100%** | **✓ CONSENSUS ACHIEVED** |
| Patient exposure, temperature control, secondary survey | **100%** | **100%** | **✓ CONSENSUS ACHIEVED** |
| Ground transfer; priority to advanced center | **75%** | **100%** | **✓ CONSENSUS ACHIEVED** |
| Aeromedical evacuation if distance >150 km | **100%** | **100%** | **✓ CONSENSUS ACHIEVED** |
| Documentation of care provided | **100%** | **100%** | **✓ CONSENSUS ACHIEVED** |

**PHASE 2 – EMERGENCY CARE**

*Emergency medicine subgroup (n = 4 experts + 1 moderator)*

**Q3 —** *What is the best up-to-date protocol for managing a patient with a head injury in a low-complexity emergency department (without CT)?*

Algorithm produced: Algorithm 3 – Figure 5

| **Recommendation item voted on** | **Subgroup (100%)** | **Plenary (94% (16/17))** | **Consensus** |
| --- | --- | --- | --- |
| Handoff with prehospital team; PPE; TBI triage checklist; trauma/resuscitation room | **100%** | **100%** | **✓ CONSENSUS ACHIEVED** |
| Telemedicine protocol with neurosurgery if available | **100%** | **100%** | **✓ CONSENSUS ACHIEVED** |
| Hemorrhage assessment and control; notify surgery/orthopedics as appropriate | **100%** | **100%** | **✓ CONSENSUS ACHIEVED** |
| Airway patency assessment | **80%** | **R1: 82% → R2: 94%** | **✓ CONSENSUS ACHIEVED** |
| GCS assessment; jaw-thrust/supraglottic devices for GCS ≤8; RSI if trained | **80%** | **100%** | **✓ CONSENSUS ACHIEVED** |
| SpO₂ assessment; supplemental oxygen if SpO₂ <94% | **80%** | **94% (16/17)** | **✓ CONSENSUS ACHIEVED** |
| SMR criteria and implementation | **100%** | **94% (16/17)** | **✓ CONSENSUS ACHIEVED** |
| Thoracic injury assessment; corrective measures if indicated | **100%** | **100%** | **✓ CONSENSUS ACHIEVED** |
| Ventilatory failure management; BVM or RSI targeting RR 10–20 and SpO₂ >94% | **100%** | **94% (16/17)** | **✓ CONSENSUS ACHIEVED** |
| SBP monitoring; IV/IO access; fluid therapy for SBP <90 mmHg (max 2L crystalloids) | **100%** | **100%** | **✓ CONSENSUS ACHIEVED** |
| TXA administration for mild-moderate TBI within 3 hours | **100%** | **100%** | **✓ CONSENSUS ACHIEVED** |
| Capillary glucose; dextrose for hypoglycemia; target 110–180 mg/dL | **100%** | **94% (16/17)** | **✓ CONSENSUS ACHIEVED** |
| Anticonvulsant prophylaxis if indicated; seizure management | **100%** | **100%** | **✓ CONSENSUS ACHIEVED** |
| Motor deficit and pupil assessment; urgent referral criteria | **100%** | **100%** | **✓ CONSENSUS ACHIEVED** |
| Non-invasive neuromonitoring if available (ONSD, pupillometry) | **80%** | **94% (16/17)** | **✓ CONSENSUS ACHIEVED** |
| Pain assessment and analgesia | **80%** | **100%** | **✓ CONSENSUS ACHIEVED** |
| Vasopressors or HTS if SBP remains <90 mmHg despite fluid therapy | **100%** | **R1: 88% → R2: 100%** | **✓ CONSENSUS ACHIEVED** |
| Neuroprotection goals maintenance (Table 12) | **80%** | **100%** | **✓ CONSENSUS ACHIEVED** |
| Temperature control; secondary survey | **100%** | **100%** | **✓ CONSENSUS ACHIEVED** |
| CT criteria assessment (Table 2) and urgent referral if indicated | **100%** | **94% (16/17)** | **✓ CONSENSUS ACHIEVED** |
| Denver Criteria (Table 10) for CT angiography need assessment | **100%** | **94% (16/17)** | **✓ CONSENSUS ACHIEVED** |
| Ground transfer; aeromedical evacuation if distance >150 km | **80%** | **94% (16/17)** | **✓ CONSENSUS ACHIEVED** |

**Q4 and Q5 —** *What is the best up-to-date protocol for managing a patient with a head injury in a medium and high-complexity emergency department?*

Algorithm produced: Algorithm 4 – Figure 6

| **Recommendation item voted on** | **Subgroup (75%)** | **Plenary (100%)** | **Consensus** |
| --- | --- | --- | --- |
| Handoff with prehospital team; PPE; TBI triage checklist | **100%** | **100%** | **✓ CONSENSUS ACHIEVED** |
| Telemedicine protocol with neurosurgery if available | **80%** | **100%** | **✓ CONSENSUS ACHIEVED** |
| Hemorrhage assessment and control | **100%** | **94% (16/17)** | **✓ CONSENSUS ACHIEVED** |
| Airway patency assessment | **75%** | **100%** | **✓ CONSENSUS ACHIEVED** |
| GCS and consciousness assessment; RSI for GCS ≤8 if indicated | **100%** | **100%** | **✓ CONSENSUS ACHIEVED** |
| SpO₂ monitoring; supplemental oxygen if SpO₂ <94% | **100%** | **94% (16/17)** | **✓ CONSENSUS ACHIEVED** |
| SMR criteria assessment and implementation | **100%** | **100%** | **✓ CONSENSUS ACHIEVED** |
| Thoracic injury assessment and management | **80%** | **R1: 76% → R2: 94%** | **✓ CONSENSUS ACHIEVED** |
| Ventilatory failure management with BVM or RSI | **75%** | **100%** | **✓ CONSENSUS ACHIEVED** |
| SBP monitoring and fluid/vasopressor therapy for SBP <90 mmHg | **100%** | **94% (16/17)** | **✓ CONSENSUS ACHIEVED** |
| TXA for mild-moderate TBI within 3 hours; lyophilized plasma/HTS if available | **75%** | **100%** | **✓ CONSENSUS ACHIEVED** |
| Capillary glucose management; target 110–180 mg/dL | **100%** | **100%** | **✓ CONSENSUS ACHIEVED** |
| Anticonvulsant prophylaxis; seizure management | **80%** | **100%** | **✓ CONSENSUS ACHIEVED** |
| Neurological assessment; urgent surgical/referral criteria | **75%** | **100%** | **✓ CONSENSUS ACHIEVED** |
| Non-invasive neuromonitoring (ONSD, TCD, pupillometry) | **80%** | **100%** | **✓ CONSENSUS ACHIEVED** |
| Biomarker assessment (GFAP, UCH-L1) if available | **80%** | **100%** | **✓ CONSENSUS ACHIEVED** |
| Pain assessment and analgesia | **100%** | **100%** | **✓ CONSENSUS ACHIEVED** |
| Vasopressors or HTS if SBP <90 mmHg persists | **100%** | **100%** | **✓ CONSENSUS ACHIEVED** |
| Neuroprotection goals maintenance | **75%** | **100%** | **✓ CONSENSUS ACHIEVED** |
| Temperature control; secondary survey | **100%** | **100%** | **✓ CONSENSUS ACHIEVED** |
| Brain CT with ABCDE-Z interpretation and neurosurgical criteria assessment | **100%** | **R1: 82% → R2: 100%** | **✓ CONSENSUS ACHIEVED** |
| Denver Criteria for CT angiography; neurosurgery consultation | **100%** | **100%** | **✓ CONSENSUS ACHIEVED** |

**PHASE 3 – NEUROLOGICAL SURGERY**

*Neurosurgery subgroup (n = 5 experts + 1 moderator)*

**Q6 —** *What is the best protocol for managing a patient requiring urgent surgery in a facility WITHOUT neurosurgical services but with general surgery and CT?*

Algorithm produced: Algorithm 5 – Figure 8

| **Recommendation item voted on** | **Subgroup (80%)** | **Plenary (100%)** | **Consensus** |
| --- | --- | --- | --- |
| Neurosurgical criteria assessment for prompt referral after stabilization | **100%** | **100%** | **✓ CONSENSUS ACHIEVED** |
| Neurological exam, CT interpretation (ABCDE-Z), non-invasive neuromonitoring, biomarkers (Table 11) | **80%** | **100%** | **✓ CONSENSUS ACHIEVED** |
| Severe TBI Code activation for immediate referral when surgical criteria met | **75%** | **94% (16/17)** | **✓ CONSENSUS ACHIEVED** |
| Hyperosmolar therapy (7.5% HTS 3-4 ml/kg q6h), anticonvulsant prophylaxis, sedation/analgesia while awaiting referral | **100%** | **100%** | **✓ CONSENSUS ACHIEVED** |
| Neuroprotection goals maintenance (Table 12) | **100%** | **94% (16/17)** | **✓ CONSENSUS ACHIEVED** |
| Continuous vital signs and neurological monitoring; repeat CT for deterioration | **100%** | **100%** | **✓ CONSENSUS ACHIEVED** |
| Telemedicine protocol with neurosurgery | **100%** | **94% (16/17)** | **✓ CONSENSUS ACHIEVED** |
| Aeromedical evacuation if distance >150 km | **100%** | **94% (16/17)** | **✓ CONSENSUS ACHIEVED** |
| Referral for specialty follow-up if intracranial bleeding without surgical criteria | **75%** | **100%** | **✓ CONSENSUS ACHIEVED** |
| Non-invasive neuromonitoring every 6–8 hours minimum during observation | **100%** | **100%** | **✓ CONSENSUS ACHIEVED** |
| Repeat CT and surgical criteria reassessment for neurological deterioration | **100%** | **R1: 76% → R2: 94%** | **✓ CONSENSUS ACHIEVED** |
| Neuroprotection goals maintenance | **75%** | **100%** | **✓ CONSENSUS ACHIEVED** |
| Exploratory trephination by non-neurosurgical personnel NOT recommended | **100%** | **94% (16/17)** | **✓ CONSENSUS ACHIEVED** |
| Brain Death Diagnostic Protocol if bilateral non-reactive mydriasis and absent brainstem reflexes | **100%** | **100%** | **✓ CONSENSUS ACHIEVED** |

**Q7 —** *What is the best protocol for managing a patient requiring urgent surgery in a facility WITH neurosurgery but WITHOUT ICU?*

Algorithm produced: Algorithm 6 – Figure 9

| **Recommendation item voted on** | **Subgroup (75%)** | **Plenary (100%)** | **Consensus** |
| --- | --- | --- | --- |
| Immediate neurosurgery notification after stabilization | **100%** | **94% (16/17)** | **✓ CONSENSUS ACHIEVED** |
| Neurological exam, CT, non-invasive neuromonitoring, biomarkers for surgical criteria (Table 11) | **80%** | **100%** | **✓ CONSENSUS ACHIEVED** |
| Severe TBI Code for immediate referral to center with neurosurgery + ICU | **100%** | **94% (16/17)** | **✓ CONSENSUS ACHIEVED** |
| Immediate referral if arrival at referral site within 4 hours from injury | **100%** | **100%** | **✓ CONSENSUS ACHIEVED** |
| Neurosurgical intervention if arrival >4 hours: craniotomy, decompressive craniectomy, ICP monitoring placement | **80%** | **100%** | **✓ CONSENSUS ACHIEVED** |
| Telemedicine protocol with ICU if available | **100%** | **94% (16/17)** | **✓ CONSENSUS ACHIEVED** |
| Aeromedical evacuation if distance >150 km | **80%** | **100%** | **✓ CONSENSUS ACHIEVED** |
| Postoperative monitoring in OR/resuscitation area until ICU transfer | **80%** | **94% (16/17)** | **✓ CONSENSUS ACHIEVED** |
| Continuous neurological monitoring; control CT for pupillary abnormality or expanding injury | **100%** | **R1: 82% → R2: 94%** | **✓ CONSENSUS ACHIEVED** |
| Invasive and non-invasive neuromonitoring for postoperative follow-up | **80%** | **100%** | **✓ CONSENSUS ACHIEVED** |

**Q8 —** *What is the best protocol for managing a patient requiring urgent surgery in a fully equipped facility (neurosurgery + ICU)?*

Algorithm produced: Algorithm 7 – Figure 10

| **Recommendation item voted on** | **Subgroup (80%)** | **Plenary (100%)** | **Consensus** |
| --- | --- | --- | --- |
| Surgical indications table (Table 11) — criteria and thresholds | **80%** | **94% (16/17)** | **✓ CONSENSUS ACHIEVED** |
| Surgical intervention as indicated: craniotomy, decompressive craniectomy, ICP/PbtO₂ monitoring | **100%** | **100%** | **✓ CONSENSUS ACHIEVED** |
| Invasive ICP monitoring placement when indicated | **100%** | **100%** | **✓ CONSENSUS ACHIEVED** |
| Postoperative ICU admission with multimodal neuromonitoring | **75%** | **100%** | **✓ CONSENSUS ACHIEVED** |
| Neuroprotection bundle: SBP, SpO₂, ICP, CPP, temperature, glucose targets | **80%** | **100%** | **✓ CONSENSUS ACHIEVED** |
| Telemedicine support for complex decision-making | **100%** | **100%** | **✓ CONSENSUS ACHIEVED** |
| Continuous assessment for reintervention criteria | **100%** | **100%** | **✓ CONSENSUS ACHIEVED** |

**PHASE 4 – INTENSIVE CARE**

*Intensive care subgroup (n = 5 experts + 1 moderator)*

**Q9 & Q10 —** *What is the best protocol for managing TBI in a postoperative recovery area or intermediate care unit (without full ICU)?*

Algorithm produced: Algorithm 8 – Figure 11

| **Recommendation item voted on** | **Subgroup (100%)** | **Plenary (94% (16/17))** | **Consensus** |
| --- | --- | --- | --- |
| ICU admission criteria (Table 14) | **100%** | **94% (16/17)** | **✓ CONSENSUS ACHIEVED** |
| ICU-level supportive interventions list (Table 15) | **100%** | **100%** | **✓ CONSENSUS ACHIEVED** |
| Haemodynamic, ventilatory and metabolic goals (Tables 9, 12) | **75%** | **100%** | **✓ CONSENSUS ACHIEVED** |
| Hemoglobin target ≥9 g/dL; transfusion strategy | **100%** | **R1: 88% → R2: 94%** | **✓ CONSENSUS ACHIEVED** |
| Anticonvulsant prophylaxis (Table 9) | **75%** | **94% (16/17)** | **✓ CONSENSUS ACHIEVED** |
| Non-invasive neuromonitoring: ONSD, TCD, pupillometry every 6–8 hours | **100%** | **94% (16/17)** | **✓ CONSENSUS ACHIEVED** |
| Repeat CT for neurological deterioration or non-invasive monitoring changes | **75%** | **100%** | **✓ CONSENSUS ACHIEVED** |
| Vasopressors for MAP/CPP targets if indicated | **100%** | **100%** | **✓ CONSENSUS ACHIEVED** |
| Nutritional support and metabolic management | **100%** | **100%** | **✓ CONSENSUS ACHIEVED** |
| Nutritional support and metabolic management | **100%** | **100%** | **✓ CONSENSUS ACHIEVED** |
| Brain Death Diagnostic Protocol if criteria met | **80%** | **100%** | **✓ CONSENSUS ACHIEVED** |
| Telemedicine consultation for ICU guidance | **80%** | **100%** | **✓ CONSENSUS ACHIEVED** |

**Q11 —** *What is the best protocol for managing TBI in a full-capability intensive care unit?*

Algorithm produced: Algorithm 9 – Figure 12

| **Recommendation item voted on** | **Subgroup (80%)** | **Plenary (100%)** | **Consensus** |
| --- | --- | --- | --- |
| Neuroprotective goals (Table 12) — all parameters | **100%** | **94% (16/17)** | **✓ CONSENSUS ACHIEVED** |
| ICP: maintain <22 mmHg at all times; waveform analysis as adjunct only | **100%** | **100%** | **✓ CONSENSUS ACHIEVED** |
| PbtO₂: target >20 mmHg per SIBICC | **100%** | **100%** | **✓ CONSENSUS ACHIEVED** |
| CPP: 60–70 mmHg, adjusted by autoregulatory status (MAP Challenge — Rosenthal/SIBICC) | **75%** | **100%** | **✓ CONSENSUS ACHIEVED** |
| Neuroprotection bundle: hemoglobin ≥9 g/dL, SpO₂ >94%, glucose 110–180, T° 36–37.5°C | **100%** | **100%** | **✓ CONSENSUS ACHIEVED** |
| Indications for invasive ICP + PbtO₂ monitoring (Table 16) | **100%** | **100%** | **✓ CONSENSUS ACHIEVED** |
| Invasive neuromonitoring goals and interventions (Table 13) | **100%** | **94% (16/17)** | **✓ CONSENSUS ACHIEVED** |
| Non-invasive neuromonitoring protocol (ONSD, TCD, pupillometry) | **100%** | **100%** | **✓ CONSENSUS ACHIEVED** |
| EEG monitoring for subclinical seizure detection if available | **100%** | **100%** | **✓ CONSENSUS ACHIEVED** |
| Nutritional support; metabolic and endocrine management | **100%** | **100%** | **✓ CONSENSUS ACHIEVED** |
| RASS sedation scale use and target (RASS −2 to −3 standard; −5 burst suppression) | **80%** | **100%** | **✓ CONSENSUS ACHIEVED** |
| DVT prophylaxis; infectious complication prevention | **80%** | **100%** | **✓ CONSENSUS ACHIEVED** |
| Brain Death Diagnostic Protocol; TCD as complementary test if prior acoustic window confirmed | **80%** | **94% (16/17)** | **✓ CONSENSUS ACHIEVED** |
