# Supplementary material for: Second edition of the recommendations from the Colombian consensus committee for the management of traumatic brain injury in the prehospital setting, emergency department, surgery, and intensive care (Beyond one option for treatment of traumatic brain injury: A stratified protocol [BOOTStraP])
Source: Brain Spine. 2026 Apr 12;6:106046. doi: 10.1016/j.bas.2026.106046 (PMC13101607; doi:10.1016/j.bas.2026.106046)
Supplement: Multimedia component 2 [file mmc2.docx]

**Supplementary material S2**

**ALGORITHM 1. Management algorithm for patients with TBI in Basic Emergency Transport - BET**

1. Perform the initial approach using personal protective equipment, establishing a safety perimeter and identifying potential risk factors.
2. Initiate the telemedicine protocol with the emergency department if available.
3. Assess for massive hemorrhage and/or life-threatening traumatic amputation. In case of massive hemorrhage, initiate the hemorrhage control protocol according to the bleeding site, beginning with direct pressure and escalating measures until control is achieved (Figure 3).
4. In the absence of massive hemorrhage, assess airway patency by early identification of signs of airway obstruction such as foreign bodies, facial or mandibular fractures, and/or tracheal/laryngeal injuries.
5. Simultaneously, determine the level of consciousness using the Alert, Verbal, Painful, Unresponsive (AVPU) scale or the Glasgow Coma Scale (GCS) if trained to use it (Supplementary material S2). In case of unresponsiveness or a GCS ≤ 8, perform the jaw-thrust maneuver as the preferred method to open the airway. Additionally, you may use supraglottic devices such as oropharyngeal or nasopharyngeal airways (in the absence of complex facial trauma) and laryngeal masks, if available.
6. Once the airway is patent, assess peripheral oxygen saturation (SpO₂) using a pulse oximeter if available. If SpO₂ is < 94%, administer supplemental oxygen early.
7. Assess whether spinal motion restriction (SMR) criteria are indicated (Table 3). If indicated, implement the corresponding measures, including placement of a rigid cervical collar, log-roll mobilization by 3–4 providers, maintenance of neutral longitudinal body alignment, and use of a long spine board, lateral head immobilizers, and cross-strapping with 3 or 4 anchor points. In cases of penetrating cervical injuries, cervical collars are contraindicated.
8. Assess ventilation and the presence of signs suggesting life-threatening thoracic injuries through inspection, palpation, and auscultation of the chest. If identified, initiate corrective measures immediately (Supplementary material S2), provided they do not delay transfer to the emergency department.
9. Assess for ventilatory failure (respiratory rate < 10 or > 30 breaths per minute and/or SpO₂ < 90%). In the presence of ventilatory failure, initiate bag-valve-mask (BVM) ventilation targeting a respiratory rate of 10–20 breaths per minute and SpO₂ > 94%. If these targets are not met, consider advanced airway management by trained personnel using rapid sequence intubation medications (Table 4), provided the necessary training and devices are available and transfer to the nearest emergency center is not delayed.
10. Assess for a radial pulse or measure systolic blood pressure (SBP) non-invasively if available. In the absence of a radial pulse or SBP < 90 mmHg, attempt peripheral venous access if resources and training are available; if not possible or unavailable, consider intraosseous access if the device and corresponding training are available, and initiate fluid therapy for hypotension. Administer crystalloids (normal saline or lactated Ringer's) as a 250 ml bolus, reassessing the radial pulse after each bolus, and repeat as needed until a palpable radial pulse and/or SBP > 90 mmHg are achieved, with a maximum total volume of 2 liters. Reassess for a radial pulse every 5 minutes. If vascular access is unavailable, expedite transfer to the care center.
11. Once adequate ventilation is ensured and a palpable radial pulse is established, check capillary blood glucose if a glucometer is available. In case of hypoglycemia (capillary glucose < 70 mg/dL), administer dextrose as a bolus (15–20 g) and repeat capillary glucose measurement after 5 minutes. The target capillary glucose range is 110–180 mg/dL.
12. Complete the neurological assessment performed earlier (AVPU or GCS) by checking pupillary response and verifying whether the patient presents weakness, lack of movement, or loss of sensation on one side of the body, especially in the arms or legs. Unresponsive patients, those with GCS ≤ 8, weakness or lack of movement on one side of the body, and/or a pupillary size difference > 2 mm should be urgently transferred to the appropriate care center.
13. Supplement the neurological assessment with near-infrared spectroscopy for detection of intracranial hematomas if the device is available. If the result is positive, consider that the patient has an intracranial hematoma and prioritize urgent transfer to a center with computed tomography, neurosurgery, and intensive care unit (ICU) availability when possible. During transport, adhere more strictly to SBP and SpO₂ targets.
14. Be alert for seizures. If seizures occur, initiate immediate management with midazolam 10 mg IM or diazepam 10 mg IV, depending on access and resource availability.
15. Assess whether the patient is in pain. If so, administer analgesia with dipyrone 1–2 g IV, paracetamol 1 g IV, or acetaminophen 1 g PO, depending on availability.
16. Expose the patient to complete the physical examination and control body temperature, preventing hypothermia through the use of sheets or blankets. Then, perform the secondary survey, consisting of a systematic head-to-toe examination to identify other injuries, verify vital signs, and obtain a brief history, provided it does not delay transfer.
17. Once stabilization maneuvers are completed, initiate ground transfer to a care center. Whenever possible, prioritize transfer to an advanced center with neurosurgery, computed tomography, and ICU availability, especially for unresponsive patients, those with GCS ≤ 8, weakness or lack of movement on one side of the body, pupillary size difference > 2 mm, and/or a positive near-infrared spectroscopy result. If this is not possible, transfer to the nearest emergency service with care capacity.
18. Consider aeromedical evacuation if available, if the distance is greater than 150 km from the accident site to the nearest advanced care center with neurosurgery, tomography, and ICU availability.
19. Immediately document the care provided in physical or digital format, depending on the available format.

**ALGORITHM 2. Management algorithm for patients with TBI in Advanced Emergency Transport - AET.**

1. Perform the initial approach using personal protective equipment, establishing a safety perimeter and identifying potential risk factors.
2. Initiate the telemedicine protocol with the emergency department if available.
3. Assess for massive hemorrhage and/or life-threatening traumatic amputation. In case of massive hemorrhage, initiate the hemorrhage control protocol according to the bleeding site, beginning with direct pressure and escalating measures until control is achieved (Figure 3).
4. In the absence of massive hemorrhage, assess airway patency by early identification of signs of airway obstruction such as foreign bodies, facial or mandibular fractures, and/or tracheal/laryngeal injuries.
5. Simultaneously, determine the level of consciousness using the Glasgow Coma Scale (GCS) (Supplementary Material 2). In case of GCS ≤ 8, perform the jaw-thrust maneuver as the preferred method to open the airway. Additionally, you may use supraglottic devices such as oropharyngeal or nasopharyngeal airways (in the absence of complex facial trauma) and laryngeal masks, or consider advanced airway management with orotracheal intubation using rapid sequence intubation (Table 4).
6. Once the airway is patent, assess peripheral oxygen saturation (SpO₂) using a pulse oximeter if available. If SpO₂ is < 94%, administer supplemental oxygen early.
7. Assess whether spinal motion restriction (SMR) criteria are indicated (Table 3). If indicated, implement the corresponding measures, including placement of a rigid cervical collar, log-roll mobilization by 3–4 providers, maintenance of neutral longitudinal body alignment, and use of a long spine board, lateral head immobilizers, and cross-strapping with 3 or 4 anchor points. In cases of penetrating cervical injuries, cervical collars are contraindicated.
8. Assess ventilation and the presence of signs suggesting life-threatening thoracic injuries through inspection, palpation, and auscultation of the chest. If identified, initiate corrective measures immediately (Supplementary Material 2), provided they do not delay transfer to the emergency department.
9. Assess for ventilatory failure (respiratory rate < 10 or > 30 breaths per minute and/or SpO₂ < 90%). In the presence of ventilatory failure, initiate bag-valve-mask (BVM) ventilation targeting a respiratory rate of 10–20 breaths per minute and SpO₂ > 94%. If these targets are not met, consider advanced airway management by trained personnel using rapid sequence intubation medications (Table 4), provided the necessary training and devices are available and transfer to the nearest emergency center is not delayed.
10. Measure systolic blood pressure (SBP) non-invasively. If SBP is < 90 mmHg, attempt peripheral venous access if resources and training are available; if not possible or unavailable, consider intraosseous access if the device and corresponding training are available, and initiate fluid therapy for hypotension. Administer crystalloids (normal saline or lactated Ringer's) as a 250 ml bolus, reassessing SBP and repeating as needed until SBP > 90 mmHg is achieved, with a maximum total volume of 2 liters. Reassess SBP every 5 minutes. If vascular access is unavailable, expedite transfer to the care center.
11. Administer tranexamic acid (TXA) for prevention of intracranial hemorrhagic lesion expansion, indicated only in mild-to-moderate TBI within the first 3 hours post-trauma, at a dose of 1 g in 100 ml of normal saline as a bolus over 10 minutes, followed by an infusion of 1 g in 500 ml of normal saline over 8 hours. Consider the use of lyophilized plasma if available and/or 3% hypertonic saline solution (HTS) at a dose of 3–4 ml/kg for patient resuscitation.
12. Once adequate ventilation is ensured and SBP > 90 mmHg is established, check capillary blood glucose if a glucometer is available. In case of hypoglycemia (capillary glucose < 70 mg/dL), administer dextrose as a bolus (15–20 g) and repeat capillary glucose measurement at 5 and 30 minutes. The target capillary glucose range is 110–180 mg/dL.
13. Complete the neurological assessment performed earlier (GCS) by checking pupillary response and verifying whether the patient presents seizures; if so, initiate immediate management with diazepam 10 mg IV/IM or midazolam 10 mg IV/IM depending on availability. In the absence of seizures but with evident depressed skull fracture or open cranial trauma with exposed brain tissue, initiate early anticonvulsant prophylaxis with available medications.
14. Verify the presence of weakness, lack of movement, or loss of sensation on one side of the body, especially in the arms or legs. Patients with GCS ≤ 8, weakness or lack of movement on one side of the body, and/or a pupillary size difference > 2 mm should be urgently transferred to the appropriate care center.
15. Supplement the neurological assessment with near-infrared spectroscopy for detection of intracranial hematomas if the device is available. If the result is positive, consider that the patient has an intracranial hematoma and prioritize urgent transfer to a center with computed tomography, neurosurgery, and intensive care unit (ICU) availability when possible. During transport, adhere more strictly to SBP and SpO₂ targets.
16. Perform non-invasive neuromonitoring (if available): optic nerve sheath diameter (ONSD) measurement by ultrasonography and digital pupillometry. If one or more positive results are obtained (ONSD ≥ 6 mm, or digital pupillometry with NPi < 3, anisocoria > 2 mm, or unilaterally decreased maximum constriction velocity [MCV]), it is recommended to initiate 3% hypertonic saline solution at a dose of 3–4 ml/kg (if not already started) and prioritize urgent transfer to a center with computed tomography, neurosurgery, and ICU availability when possible. During transport, adhere more strictly to SBP and SpO₂ targets.
17. Assess whether the patient is in pain. If so, administer analgesia with dipyrone 1–2 g IV, paracetamol 1 g IV, or acetaminophen 1 g PO, depending on availability.
18. Expose the patient to complete the physical examination and control body temperature, preventing hypothermia through the use of sheets or blankets. Then, perform the secondary survey, consisting of a systematic head-to-toe examination to identify other injuries, verify vital signs, and obtain a brief history, provided it does not delay transfer.
19. Once stabilization maneuvers are completed, initiate ground transfer to a care center. Whenever possible, prioritize transfer to an advanced center with neurosurgery, computed tomography, and ICU availability, especially for patients with GCS ≤ 8, weakness or lack of movement on one side of the body, pupillary size difference > 2 mm, positive near-infrared spectroscopy results, and/or positive non-invasive neuromonitoring findings. If this is not possible, transfer to the nearest emergency service with care capacity.
20. Consider aeromedical evacuation if available, if the distance is greater than 150 km from the accident site to the nearest advanced care center with neurosurgery, tomography, and ICU availability.
21. Immediately document the care provided in physical or digital format, depending on the available format.

**ALGORITHM 3. Management algorithm for patients with TBI in Low-Complexity EDs without CT.**

1. Perform an effective handoff with the prehospital care team (if applicable) and use personal protective equipment.
2. Execute the TBI triage checklist, prioritizing medical care in the trauma/resuscitation room if necessary.
3. Initiate a telemedicine protocol with neurosurgery if available.
4. Assess for massive hemorrhage and/or life-threatening traumatic amputation. In case of massive hemorrhage, initiate the hemorrhage control protocol according to the bleeding site, beginning with direct pressure and escalating measures until control is achieved (Figure 3). Immediately notify the general surgery and/or orthopedics services as appropriate.
5. In the absence of massive hemorrhage, assess airway patency by early identification of signs of airway obstruction such as foreign bodies, facial or mandibular fractures, and/or tracheal/laryngeal injuries.
6. Simultaneously, determine the level of consciousness using the Glasgow Coma Scale (GCS) (Supplementary Material 2). In case of GCS ≤ 8, perform the jaw-thrust maneuver as the preferred method to open the airway. Additionally, you may use supraglottic devices such as oropharyngeal or nasopharyngeal airways (in the absence of complex facial trauma) and laryngeal masks, or consider advanced airway management with orotracheal intubation using rapid sequence intubation (Table 4).
7. In patients with mild TBI, consider obtaining biomarkers if available.
8. Once the airway is patent, assess peripheral oxygen saturation (SpO₂) using a pulse oximeter if available. If SpO₂ is < 94%, administer supplemental oxygen early.
9. Assess whether spinal motion restriction (SMR) criteria are indicated (Table 3). If indicated, implement the corresponding measures, including placement of a rigid cervical collar, log-roll mobilization, and maintenance of neutral longitudinal body alignment. In cases of penetrating cervical injuries, cervical collars are contraindicated. Maintain the rigid cervical collar until cervical injury is ruled out by imaging studies.
10. Assess ventilation and the presence of signs suggesting life-threatening thoracic injuries through inspection, palpation, and auscultation of the chest. If identified, initiate corrective measures immediately (Supplementary Material 2) and notify the relevant specialties: general surgery, vascular surgery, and/or anesthesiology if available.
11. Assess for ventilatory failure (respiratory rate < 10 or > 30 breaths per minute and/or SpO₂ < 90%). In the presence of ventilatory failure, initiate bag-valve-mask (BVM) ventilation targeting a respiratory rate of 10–20 breaths per minute and SpO₂ > 94%. If these targets are not met, consider advanced airway management by trained personnel using rapid sequence intubation medications (Table 4). Determine considerations for advanced airway management (Table 5).
12. Measure systolic blood pressure (SBP) non-invasively. If SBP is < 90 mmHg, obtain peripheral venous access; if not possible, consider intraosseous access if the device and corresponding training are available, and initiate fluid therapy for hypotension. Administer crystalloids (normal saline or lactated Ringer's) as a 250 ml bolus, reassessing SBP and repeating as needed until SBP > 90 mmHg is achieved, with a maximum total volume of 2 liters. Reassess SBP every 5 minutes.
13. Administer tranexamic acid (TXA) for prevention of intracranial hemorrhagic lesion expansion, indicated only in mild-to-moderate TBI within the first 3 hours post-trauma, at a dose of 1 g in 100 ml of normal saline as a bolus over 10 minutes, followed by an infusion of 1 g in 500 ml of normal saline over 8 hours. Consider balanced hemostatic resuscitation if available. Evaluate the need for early blood transfusion in a 1:1:1 ratio (red blood cells:plasma:platelets); use TASH or ABC scores, lactate > 2.5 mg/dL, or base excess > -6 to consider transfusion. If hypotension persists, administer vasopressors (Table 7) or hypertonic solutions (Table 8).
14. Perform a trauma ultrasound (FAST / E-FAST) if available and assess for signs of pelvic instability. If positive for free fluid, pneumothorax, or suspected pelvic fracture (apply a pelvic binder or sheet wrap if identified), transfer to surgery or refer to a higher-complexity center for definitive management.
15. Once adequate ventilation is ensured and SBP > 90 mmHg is established, check capillary blood glucose. In case of hypoglycemia (capillary glucose < 70 mg/dL), administer dextrose as a bolus (15–20 g) and repeat capillary glucose measurement at 5 and 30 minutes. The target capillary glucose range is 110–180 mg/dL.
16. Complete the neurological assessment performed earlier (GCS) by checking pupillary response and verifying whether the patient presents seizures; if so, initiate immediate management with diazepam 10 mg IV/IM or midazolam 10 mg IV/IM depending on availability. If seizures persist after using 2 doses of benzodiazepines, initiate Levetiracetam 2 g IV or Phenytoin 15–20 mg/kg IV or Valproic Acid 10–15 mg/kg IV by infusion.
17. In the absence of seizures but with evident depressed skull fracture or open cranial trauma with exposed brain tissue, initiate early anticonvulsant prophylaxis with available medications.
18. Assess whether the patient is in pain. If so, administer analgesia with dipyrone 1–2 g IV, paracetamol 1 g IV, or acetaminophen 1 g PO, depending on availability.
19. Verify the presence of weakness, lack of movement, or loss of sensation on one side of the body, especially in the arms or legs. In patients with GCS ≤ 8, weakness or lack of movement on one side of the body, and/or a pupillary size difference > 2 mm, initiate 3% hypertonic saline solution at a dose of 3–4 ml/kg (Table 8) and arrange urgent transfer to a center with tomography and neurosurgery availability.
20. Supplement the neurological assessment with near-infrared spectroscopy for detection of intracranial hematomas if the device is available. If the result is positive, consider that the patient has an intracranial hematoma and prioritize urgent transfer to a center with computed tomography, neurosurgery, and intensive care unit (ICU) availability when possible. During transport, adhere more strictly to SBP and SpO₂ targets.
21. Perform non-invasive neuromonitoring (if available): optic nerve sheath diameter (ONSD) measurement by ultrasonography, digital pupillometry, and transcranial Doppler. If one or more positive results are obtained (ONSD ≥ 6 mm, or digital pupillometry with NPi < 3, anisocoria > 2 mm, unilaterally decreased maximum constriction velocity [MCV], or transcranial Doppler [TCD] evidence of pulsatility index [PI] > 1.3, end-diastolic velocity below 20 cm/sec), it is recommended to initiate 3% hypertonic saline solution at a dose of 3–4 ml/kg (if not already started) and prioritize urgent transfer to a center with computed tomography, neurosurgery, and ICU availability when possible. During transport, adhere more strictly to SBP and SpO₂ targets.
22. Assess the presence of criteria for brain tomography (Table 2) and immediately refer to a higher-complexity center with tomography and neurosurgery availability.
23. If criteria for brain tomography are not met, evaluate the Denver Criteria (Table 10) to define the need for CT angiography in patients with suspected vascular injury. If present, immediately refer to a higher-complexity center with tomography, neurosurgery, and surgery availability.
24. If criteria for tomography, CT angiography, or immediate referral are not met, maintain observation for 4–6 hours according to institutional protocol.
25. Expose the patient to complete the physical examination and control body temperature, preventing hypothermia through the use of sheets or blankets. Then, perform the secondary survey, consisting of a systematic head-to-toe examination to identify other injuries, verify vital signs, and obtain a brief history. Request laboratory studies.
26. If during observation the patient shows signs of neurological deterioration, consider obtaining serum biomarkers. If GFAP levels > 30 pg/mL and/or UCH-L1 > 360 pg/mL (first 12 hours of injury) are present along with neurological deterioration, initiate immediate transfer to a center with tomography and neurosurgery availability.
27. Consider aeromedical evacuation if available, if the distance is greater than 150 km to the nearest advanced care center with neurosurgery, tomography, and ICU availability.
28. If no neurological deterioration, signs of expanding intracranial injury, or negative non-invasive neuromonitoring occur during the observation period, consider discharge with recommendations and warning signs.
29. Immediately document the care provided in physical or digital format, depending on the available format.
30. Perform an effective handoff of the patient to inpatient service personnel.

**ALGORITHM 4. Management algorithm for patients with TBI in Medium and High-Complexity EDs with CT.**

1. Perform an effective handoff with the prehospital care team (if applicable) and use personal protective equipment.
2. Execute the TBI triage checklist, prioritizing medical care in the trauma/resuscitation room if necessary.
3. In case of neurological alteration, immediately notify the neurosurgery service.
4. Assess for massive hemorrhage and/or life-threatening traumatic amputation. In case of massive hemorrhage, initiate the hemorrhage control protocol according to the bleeding site, beginning with direct pressure and escalating measures until control is achieved (Figure 3). Immediately notify the general surgery and/or orthopedics services as appropriate.
5. In the absence of massive hemorrhage, assess airway patency by early identification of signs of airway obstruction such as foreign bodies, facial or mandibular fractures, and/or tracheal/laryngeal injuries.
6. Simultaneously, determine the level of consciousness using the Glasgow Coma Scale (GCS) (Supplementary Material 2). In case of GCS ≤ 8, perform the jaw-thrust maneuver as the preferred method to open the airway. Additionally, you may use supraglottic devices such as oropharyngeal or nasopharyngeal airways (in the absence of complex facial trauma) and laryngeal masks, or consider advanced airway management with orotracheal intubation using rapid sequence intubation (Table 4).
7. In patients with mild TBI, consider obtaining biomarkers if available.
8. Once the airway is patent, assess peripheral oxygen saturation (SpO₂) using a pulse oximeter if available. If SpO₂ is < 94%, administer supplemental oxygen early.
9. Assess whether spinal motion restriction (SMR) criteria are indicated (Table 3). If indicated, implement the corresponding measures, including placement of a rigid cervical collar, log-roll mobilization, and maintenance of neutral longitudinal body alignment. In cases of penetrating cervical injuries, cervical collars are contraindicated. Maintain the rigid cervical collar until cervical injury is ruled out by imaging studies.
10. Assess ventilation and the presence of signs suggesting life-threatening thoracic injuries through inspection, palpation, and auscultation of the chest. If identified, initiate corrective measures immediately (Supplementary Material 2) and notify the relevant specialties: general surgery, vascular surgery, and/or anesthesiology if available.
11. Assess for ventilatory failure (respiratory rate < 10 or > 30 breaths per minute and/or SpO₂ < 90%). In the presence of ventilatory failure, initiate bag-valve-mask (BVM) ventilation targeting a respiratory rate of 10–20 breaths per minute and SpO₂ > 94%. If these targets are not met, consider advanced airway management by trained personnel using rapid sequence intubation medications (Table 4). Determine considerations for advanced airway management (Table 5).
12. Measure systolic blood pressure (SBP) non-invasively. If SBP is < 90 mmHg, obtain double peripheral venous access; if not possible, consider intraosseous access or a central venous catheter, if the device and corresponding training are available, and initiate fluid therapy for hypotension. Administer crystalloids (normal saline or lactated Ringer's) as a 250 ml bolus, reassessing SBP and repeating as needed until SBP > 90 mmHg is achieved, with a maximum total volume of 2 liters. Reassess SBP every 5 minutes.
13. Administer tranexamic acid (TXA) for prevention of intracranial hemorrhagic lesion expansion, indicated only in mild-to-moderate TBI within the first 3 hours post-trauma, at a dose of 1 g in 100 ml of normal saline as a bolus over 10 minutes, followed by an infusion of 1 g in 500 ml of normal saline over 8 hours. Consider balanced hemostatic resuscitation if available. Evaluate the need for early blood transfusion in a 1:1:1 ratio (red blood cells:plasma:platelets); use TASH or ABC scores, lactate > 2.5 mg/dL, or base excess > -6 to consider transfusion. If hypotension persists, administer vasopressors (Table 7) or hypertonic solutions (Table 8).
14. Perform a trauma ultrasound (FAST / E-FAST) if available and assess for signs of pelvic instability. If positive for free fluid, pneumothorax, or suspected pelvic fracture (apply a pelvic binder or sheet wrap if identified), transfer to surgery or refer to a higher-complexity center for definitive management.
15. Once adequate ventilation is ensured and SBP > 90 mmHg is established, check capillary blood glucose. In case of hypoglycemia (capillary glucose < 70 mg/dL), administer dextrose as a bolus (15–20 g) and repeat capillary glucose measurement at 5 and 30 minutes. The target capillary glucose range is 110–180 mg/dL.
16. Complete the neurological assessment performed earlier (GCS) by checking pupillary response and verifying whether the patient presents seizures; if so, initiate immediate management with diazepam 10 mg IV/IM or midazolam 10 mg IV/IM depending on availability. If seizures persist after using 2 doses of benzodiazepines, initiate Levetiracetam 2 g IV or Phenytoin 15–20 mg/kg IV or Valproic Acid 10–15 mg/kg IV by infusion.
17. In the absence of seizures but with evident depressed skull fracture or open cranial trauma with exposed brain tissue, initiate early anticonvulsant prophylaxis with available medications.
18. Assess whether the patient is in pain. If so, administer analgesia with dipyrone 1–2 g IV, paracetamol 1 g IV, or acetaminophen 1 g PO, depending on availability.
19. Verify the presence of weakness, lack of movement, or loss of sensation on one side of the body, especially in the arms or legs. In patients with GCS ≤ 8, weakness or lack of movement on one side of the body, and/or a pupillary size difference > 2 mm, initiate 3% hypertonic saline solution at a dose of 3–4 ml/kg (Table 8) and urgently transfer to the tomography service and notify the neurosurgery service.
20. Supplement the neurological assessment with near-infrared spectroscopy for detection of intracranial hematomas if the device is available. If the result is positive, consider that the patient has an intracranial hematoma and urgently transfer to the tomography service and notify the neurosurgery service.
21. Perform non-invasive neuromonitoring (if available): optic nerve sheath diameter (ONSD) measurement by ultrasonography, digital pupillometry, and transcranial Doppler. If one or more positive results are obtained (ONSD ≥ 6 mm, or digital pupillometry with NPi < 3, anisocoria > 2 mm, unilaterally decreased maximum constriction velocity [MCV], or transcranial Doppler [TCD] evidence of pulsatility index [PI] > 1.3, end-diastolic velocity [EDV] below 20 cm/sec), it is recommended to initiate 3% hypertonic saline solution at a dose of 3–4 ml/kg (if not already started) and expedite transfer to the tomography service. During transport, adhere more strictly to SBP and SpO₂ targets, and also consider evaluation by the ICU.
22. Assess the presence of criteria for brain tomography (Table 2) and urgently transfer to the tomography service and notify the neurosurgery service.
23. It is recommended to use the ABCDE sequence for brain CT interpretation and to evaluate surgical criteria (Table 11 and Go to Algorithm #7).
24. It is also recommended to alert the blood bank and operating room in case of a patient meeting surgical criteria.
25. If criteria for brain tomography are not met, it is recommended to evaluate the Denver Criteria (Table 10) to define the need for CT angiography in patients with suspected vascular injury, urgently transfer to the tomography service and notify the neurosurgery, general surgery, vascular surgery, and/or orthopedics services as appropriate.
26. In case of abnormal brain CT or CT angiography but without surgical criteria, perform a follow-up CT scan 6 hours after the initial scan.
27. If criteria for tomography, CT angiography, or immediate referral are not met, maintain observation for 4–6 hours according to institutional protocol, performing hourly assessments to monitor for: a drop of ≥ 2 points in the GCS, pupillary asymmetry > 2 mm, new focal deficit, non-reactive pupils, and if possible, perform non-invasive neuromonitoring every hour.
28. Expose the patient to complete the physical examination and control body temperature, preventing hypothermia through the use of sheets or blankets. Then, perform the secondary survey, consisting of a systematic head-to-toe examination to identify other injuries, verify vital signs, and obtain a brief history. Request laboratory studies.
29. If during observation the patient shows signs of neurological deterioration, consider obtaining serum biomarkers. If GFAP levels > 30 pg/mL and/or UCH-L1 > 360 pg/mL (first 12 hours of injury) are present along with neurological deterioration, perform a brain CT scan and notify the neurosurgery service for a patient with possible surgical criteria or to escalate the level of ICP treatment.
30. If no neurological deterioration, signs of expanding intracranial injury, or negative non-invasive neuromonitoring occur during the observation period, consider discharge with recommendations and warning signs.
31. Immediately document the care provided in physical or digital format, depending on the available format.
32. Perform an effective handoff of the patient to inpatient service personnel.

**ALGORITHM 5. Management algorithm for patients with TBI requiring surgery at centers without Neurosurgical Capacity**

1. After adequate stabilization and resuscitation of the patient in the emergency department, determine the presence of neurosurgical criteria for prompt referral.
2. Perform the neurological clinical examination (GCS and pupils), scalp inspection, brain CT interpretation using the ABCDE method, non-invasive neuromonitoring (optic nerve sheath diameter measurement, digital pupillometry, and transcranial Doppler) if available, and obtain biomarkers if available, to determine the presence of neurosurgical criteria (Table 11).
3. Once the need for emergency surgical intervention is determined, activate the Severe TBI Code for immediate patient referral.
4. While awaiting referral, initiate hyperosmolar therapy (7.5% HTS at a dose of 3–4 ml/kg every 6 hours) (Table 8), anticonvulsant prophylaxis (Table 9), and early sedation/analgesia in patients meeting surgical criteria.
5. Maintain baseline neuroprotection goals (Table 12).
6. Perform continuous monitoring of vital signs and neurological assessments (GCS and pupils). In case of new neurological deterioration, obtain a repeat brain CT scan.
7. Initiate a telemedicine protocol with neurosurgery if available.
8. Consider aeromedical evacuation if available, if the distance is greater than 150 km to the nearest advanced care center with neurosurgery, tomography, and ICU availability.
9. Patients who do not initially meet surgical criteria but have intracerebral bleeding or neurological warning signs should be considered for referral to a center with neurosurgery availability for specialty follow-up.
10. While awaiting referral, perform continuous monitoring of vital signs and neurological assessment (GCS and pupils). Perform non-invasive neuromonitoring (if available): optic nerve sheath diameter (ONSD) measurement by ultrasonography, digital pupillometry, and transcranial Doppler every 6–8 hours (minimum every 24 hours).
11. In the presence of signs of neurological deterioration, suspicion of expanding intracranial injury, and/or altered non-invasive neuromonitoring (ONSD ≥ 6 mm, TCD with PI > 1.3, MCA end-diastolic velocity [EDV] below 20 cm/sec, and/or digital pupillometry with NPi < 3, anisocoria > 2 mm, or unilaterally decreased MCV), reassess surgical criteria by obtaining a new brain CT scan.
12. Maintain baseline neuroprotection goals (Table 12).
13. Exploratory trephination by non-neurosurgical medical personnel is not recommended.
14. If during observation, patients develop bilateral non-reactive mydriasis and absent brainstem reflexes, initiate the Brain Death Diagnostic Protocol and notify the Organ Donation and Transplantation Network. The use of transcranial Doppler as a complementary diagnostic test is reserved for cases with a previously documented adequate acoustic window and cerebral blood flow prior to the suspicion of brain death and is not considered a requirement for confirmatory diagnosis.

**ALGORITHM 6. Management algorithm for TBI patients requiring surgery at centers with Neurosurgical but without Intensive Care Capacity.**

1. After adequate stabilization and resuscitation of the patient in the emergency department, immediately notify the neurosurgery service.
2. Perform the neurological clinical examination (GCS and pupils), scalp inspection, brain CT interpretation using the ABCDE method, perform non-invasive neuromonitoring (optic nerve sheath diameter measurement, transcranial Doppler and digital pupillometry) if available, and obtain biomarkers if available, to determine the presence of neurosurgical criteria (Table 11).
3. Once the need for emergency surgical intervention is determined, activate the Severe TBI Code for immediate patient referral to a center with neurosurgery and intensive care unit availability.
4. If arrival at the referral site is possible within the next 4 hours from the time of injury, initiate the referral process immediately.
5. If arrival at the referral site is not possible within the next 4 hours from the time of injury, perform the neurosurgical intervention as indicated (Table 11): craniotomy for hematoma drainage, decompressive craniectomy, elevation of skull fracture, repair of scalp avulsion wounds, cisternostomy, and/or placement of invasive monitoring (ventricular catheter, ICP catheter, or ICP+PbtO₂ catheter if available), provide postoperative care, and continue the referral plan for postoperative ICU.
6. Initiate a telemedicine protocol with the intensive care unit if available.
7. Consider aeromedical evacuation if available, if the distance is greater than 150 km to the nearest advanced care center with neurosurgery, tomography, and ICU availability.
8. While awaiting referral, provide postoperative monitoring in the operating room, resuscitation area, or emergency department (under the supervision of an anesthesiologist, emergency physician, intensivist, and/or neurosurgeon) until transfer to a center with ICU availability is achieved.
9. Perform continuous monitoring of vital signs and neurological examination (GCS and pupils) (without interrupting sedation/analgesia for GCS assessment). In case of pupillary abnormality or suspicion of expanding intracranial injury, perform a control CT scan to evaluate criteria for surgical reintervention.
10. Perform invasive and non-invasive neuromonitoring (if available) for postoperative follow-up: optic nerve sheath diameter (ONSD) measurement by ultrasonography, transcranial Doppler, and digital pupillometry every 6–8 hours (minimum every 24 hours). In case of abnormal invasive or non-invasive neuromonitoring, perform a control CT scan to evaluate criteria for surgical reintervention.
11. Obtain biomarkers for postoperative follow-up (if available).
12. Maintain baseline neuroprotection goals (Table 12) and invasive monitoring goals if available (Table 13).
13. While awaiting referral, initiate hyperosmolar therapy (7.5% HTS at a dose of 3–4 ml/kg every 6 hours) (Table 8), anticonvulsant prophylaxis (Table 9), prophylactic antibiotics, and maintain sedation/analgesia for RASS -5 (if required, consider neuromuscular blockade to achieve sedation goals).
14. If during observation, patients develop bilateral non-reactive mydriasis and absent brainstem reflexes, initiate the Brain Death Diagnostic Protocol and notify the Organ Donation and Transplantation Network. The use of transcranial Doppler as a complementary diagnostic test is reserved for cases with a previously documented adequate acoustic window and cerebral blood flow prior to the suspicion of brain death and is not considered a requirement for confirmatory diagnosis.

**ALGORITHM 7. Management algorithm for TBI patients requiring surgery at centers with Neurosurgical and Intensive Care Capacity.**

1. After adequate stabilization and resuscitation of the patient in the emergency department, immediately notify the neurosurgery service.
2. Perform the neurological clinical examination (GCS and pupils), scalp inspection, along with brain CT interpretation using the ABCDE method, perform non-invasive neuromonitoring (optic nerve sheath diameter measurement, transcranial Doppler, and digital pupillometry) if available, and obtain biomarkers if available, to determine the presence of neurosurgical criteria (Table 11).
3. Once the need for emergency surgical intervention is determined, notify the blood bank service and perform the neurosurgical intervention as indicated (Table 11): craniotomy for hematoma drainage, decompressive craniectomy, elevation of skull fracture, repair of scalp avulsion wounds, cisternostomy, and/or placement of invasive monitoring (ventricular catheter, ICP catheter, or ICP+PbtO₂ catheter if available), followed by transfer to the ICU for postoperative monitoring and follow-up.
4. Perform continuous monitoring of vital signs and neurological examination (GCS and pupils) (without interrupting sedation/analgesia for GCS assessment). In case of pupillary abnormality or suspicion of expanding intracranial injury, perform a control CT scan to evaluate criteria for surgical reintervention.
5. Perform invasive (Table 13) and non-invasive neuromonitoring (if available) for postoperative follow-up: optic nerve sheath diameter (ONSD) measurement by ultrasonography, transcranial Doppler, and digital pupillometry every 6–8 hours (minimum every 24 hours). In case of abnormal invasive or non-invasive neuromonitoring, perform a control CT scan to evaluate criteria for surgical reintervention.
6. Maintain baseline neuroprotection goals (Table 12) and invasive monitoring goals if available (Table 13).
7. Initiate hyperosmolar therapy (7.5% HTS at a dose of 3–4 ml/kg every 6 hours) (Table 8), anticonvulsant prophylaxis (Table 9), prophylactic antibiotics, and maintain sedation/analgesia for RASS -5 (if required, consider neuromuscular blockade to achieve sedation goals).
8. Perform a control brain CT scan within the first 24 hours postoperatively.
9. If during observation, patients develop bilateral non-reactive mydriasis and absent brainstem reflexes, initiate the Brain Death Diagnostic Protocol and notify the Organ Donation and Transplantation Network. The use of transcranial Doppler as a complementary diagnostic test is reserved for cases with a previously documented adequate acoustic window and cerebral blood flow prior to the suspicion of brain death and is not considered a requirement for confirmatory diagnosis.
10. Perform a brain MRI if diffuse axonal injury, persistent non-reactive/poorly reactive state, and/or brainstem hemorrhage are suspected.
11. Perform continuous electroencephalogram (minimum 6-hour telemetry) if non-convulsive status epilepticus or subclinical seizures are suspected.
12. Use the heatmap to guide the weaning of respiratory support, sedation/analgesia, and withdrawal of invasive monitoring, in accordance with the SIBICC protocol and neuromonitoring trends.

**ALGORITHM 8. Management algorithm for TBI patients requiring ICU-Level Care at centers without ICU but with Postoperative Recovery Area Capacity or Intermediate Care Capacity.**

1. Early define intensive care unit (ICU) admission criteria (Table 14).
2. If the patient meets ICU admission criteria, activate the Severe TBI Code for immediate referral to a center with ICU availability.
3. Initiate a telemedicine protocol with the ICU (if available).
4. Consider aeromedical evacuation if available, if the distance is greater than 150 km to the nearest advanced care center with neurosurgery, tomography, and ICU availability.
5. While awaiting referral, admit the patient to the area with the highest available monitoring capacity (intermediate care unit, postoperative recovery room, or emergency department).
6. Perform continuous monitoring of vital signs and neurological examination (GCS, pupils, and FOUR [Full Outline of UnResponsiveness] scale) without interrupting sedation/analgesia for scale assessment. In case of pupillary abnormality or suspicion of expanding intracranial injury, perform a control CT scan and notify the neurosurgery service to evaluate criteria for surgical reintervention.
7. Perform invasive (Table 13) and non-invasive neuromonitoring (if available) for postoperative follow-up: optic nerve sheath diameter (ONSD) measurement by ultrasonography, transcranial Doppler, and digital pupillometry every 6–8 hours (minimum every 24 hours). In case of abnormal invasive or non-invasive neuromonitoring, perform a control CT scan and notify the neurosurgery service to evaluate criteria for surgical reintervention.
8. Obtain biomarkers for follow-up (if available). If this is the first measurement, identify the trend to correlate with the outcome. If levels were already measured previously, compare the trend with the previous level from the ED to correlate with the outcome. The frequency of measurements will be decided by the treatment group.
9. In case of neurological deterioration or abnormal invasive/non-invasive neuromonitoring, initiate hyperosmolar therapy (7.5% HTS at a dose of 3–4 ml/kg) (Table 8).
10. If surgical reintervention criteria are not met, initiate the tiered ICP management protocol at the most appropriate level, and reevaluate after each intervention. Escalate therapy until effective or until the maximum tier is reached.
    1. Level 1: Scheduled hypertonic saline, optimize analgesia, optimize sedation, and CSF drainage if an EVD (external ventricular drain) is in place.
    2. Level 2: Neuromuscular blockade, mild hypocapnia (PaCO₂ 32–35 mmHg). Increase CPP (cerebral perfusion pressure) if autoregulation is intact.
    3. Level 3: Pentobarbital or thiopental coma. Mild hypothermia (35–36°C). Secondary decompressive craniectomy.
11. If during observation, patients develop bilateral non-reactive mydriasis and absent brainstem reflexes, initiate the Brain Death Diagnostic Protocol and notify the Organ Donation and Transplantation Network. The use of transcranial Doppler as a complementary diagnostic test is reserved for cases with a previously documented adequate acoustic window and cerebral blood flow prior to the suspicion of brain death and is not considered a requirement for confirmatory diagnosis.
12. Initiate general ICU management measures (Table 15).
13. Maintain baseline neuroprotection goals (Table 12) and invasive monitoring goals if available (Table 13).
14. Evaluate criteria for early anticonvulsant prophylaxis initiation (Table 9) and manage with benzodiazepines if a seizure is witnessed.
15. Maintain sedation/analgesia for RASS -5 and/or BIS (Bispectral Index) 40–60 (if available). Recommended analgesics include: fentanyl, remifentanil; and sedatives include: midazolam, propofol, and ketamine.
16. If the patient does not meet ICU criteria, maintain under surveillance in the emergency department or hospitalization according to neurosurgery criteria, and perform scheduled clinical neurological monitoring (GCS and pupillary assessment) during the first 24 hours.
17. Perform non-invasive neuromonitoring (if available): optic nerve sheath diameter (ONSD) measurement by ultrasonography, digital pupillometry, and transcranial Doppler every 6–8 hours (maximum every 24 hours).
18. In the presence of signs of neurological deterioration, suspicion of expanding intracranial injury, and/or altered non-invasive neuromonitoring (ONSD ≥ 6 mm, TCD with PI > 1.3, MCA end-diastolic velocity [EDV] below 20 cm/sec, and/or digital pupillometry with NPi < 3, anisocoria > 2 mm, or unilaterally decreased MCV), initiate 7.5% hyperosmolar therapy at 3–4 ml/kg, perform a new control CT scan, request neurosurgery evaluation, and reassess the presence of surgical and ICU admission criteria.

**ALGORITHM 9. Management algorithm for TBI patients requiring ICU-Level Care and ICU capacity.**

1. Early define intensive care unit (ICU) admission criteria (Table 14).
2. If the patient meets ICU admission criteria, transfer to a general ICU (with advanced equipment and trained personnel).
3. Initiate a telemedicine protocol with neuro-ICU/neurocritical care (if available).
4. In the general ICU, perform continuous monitoring of vital signs and scheduled neurological examination, including pupillary assessment, GCS, and FOUR scale (without interrupting sedation/analgesia for scale assessment). In case of pupillary abnormality or suspicion of expanding intracranial injury, perform a control CT scan and notify the neurosurgery service to evaluate criteria for surgical reintervention.
5. Perform invasive neuromonitoring if available (Table 13) and non-invasive neuromonitoring for postoperative follow-up: optic nerve sheath diameter (ONSD) measurement by ultrasonography, transcranial Doppler, and digital pupillometry every 6–8 hours (minimum every 24 hours). In case of abnormal invasive or non-invasive neuromonitoring, perform a control CT scan and notify the neurosurgery service to evaluate criteria for surgical reintervention.
6. Obtain biomarkers for follow-up (if available). If this is the first measurement, identify the trend to correlate with the outcome. If levels were already measured previously, compare the trend with the previous level from the ED to correlate with the outcome. The frequency of measurements will be decided by the treatment group.
7. In case of neurological deterioration or abnormal invasive/non-invasive neuromonitoring, initiate hyperosmolar therapy (7.5% HTS at a dose of 3–4 ml/kg) (Table 8).
8. If surgical reintervention criteria are not met, initiate the tiered ICP management protocol at the most appropriate level, and reevaluate after each intervention. Escalate therapy until effective or until the maximum tier is reached.
   1. Level 1: Scheduled hypertonic saline, optimize analgesia, optimize sedation, and CSF drainage if an EVD (external ventricular drain) is in place.
   2. Level 2: Neuromuscular blockade, mild hypocapnia (PaCO₂ 32–35 mmHg). Increase CPP (cerebral perfusion pressure) if autoregulation is intact.
   3. Level 3: Pentobarbital or thiopental coma. Mild hypothermia (35–36°C). Secondary decompressive craniectomy.
9. Initiate general ICU management measures (Table 15).
10. Maintain baseline neuroprotection goals (Table 12) and invasive monitoring goals if available (Table 13).
11. In case of abnormalities in natremia, diuresis, glycemia, or refractory hypotension, consider post-traumatic pituitary dysfunction.
12. Evaluate criteria for early anticonvulsant prophylaxis initiation (Table 9) and manage with benzodiazepines, followed by levetiracetam and lacosamide in case of seizures.
13. Maintain sedation/analgesia for RASS -5 and/or BIS (Bispectral Index) 40–60 (if available). Recommended analgesics include: fentanyl, remifentanil; and sedatives include: midazolam, propofol, and ketamine.
14. If patients develop bilateral non-reactive mydriasis and absent brainstem reflexes, initiate the Brain Death Diagnostic Protocol and notify the Transplantation Procurement Office. The use of transcranial Doppler as a complementary diagnostic test is reserved for cases with a previously documented cerebral blood flow prior to the suspicion of brain death and is not considered a requirement for confirmatory diagnosis.
15. Perform a brain MRI if diffuse axonal injury, persistent non-reactive/poorly reactive state, and/or brainstem hemorrhage are suspected.
16. Perform continuous electroencephalogram (minimum 6-hour telemetry) if non-convulsive status epilepticus or subclinical seizures are suspected.
17. Use the heatmap to guide the weaning of respiratory support, sedation/analgesia, and withdrawal of invasive monitoring, in accordance with the SIBICC protocol and neuromonitoring trends.
18. If the patient does not meet ICU criteria, maintain under surveillance in the emergency department or hospitalization according to neurosurgery criteria, and perform scheduled clinical neurological monitoring (GCS and pupillary assessment) during the first 24 hours.
19. Perform non-invasive neuromonitoring (if available): optic nerve sheath diameter (ONSD) measurement by ultrasonography, digital pupillometry, and transcranial Doppler every 6–8 hours (maximum every 24 hours).
20. In the presence of signs of neurological deterioration, suspicion of expanding intracranial injury, and/or altered non-invasive neuromonitoring (ONSD ≥ 6 mm, TCD with PI > 1.3, MCA end-diastolic velocity [EDV] below 20 cm/sec, and/or digital pupillometry with NPi < 3, anisocoria > 2 mm, or unilaterally decreased MCV), initiate 7.5% hyperosmolar therapy at 3–4 ml/kg, perform a new control CT scan, request neurosurgery evaluation, and reassess the presence of surgical and ICU admission criteria.
