# Supplementary material for: Second edition of the recommendations from the Colombian consensus committee for the management of traumatic brain injury in the prehospital setting, emergency department, surgery, and intensive care (Beyond one option for treatment of traumatic brain injury: A stratified protocol [BOOTStraP])
Source: Brain Spine. 2026 Apr 12;6:106046. doi: 10.1016/j.bas.2026.106046 (PMC13101607; doi:10.1016/j.bas.2026.106046)
Supplement: Multimedia component 3 [file mmc3.docx]

**Supplementary material S3**

**APPENDIX A.** AVPU and Glasgow Coma Scale.

**AVPU**

| **A** | Patient is **Alert.** |
| --- | --- |
| **V** | Patient responds to **Verbal** stimuli but may not be fully oriented or aware of their surroundings. They may open their eyes or respond to verbal commands. |
| **P** | Patient responds only to **Painful** stimuli such as nail bed pressure or trapezius squeeze. |
| **U** | Patient is **Unresponsive.** |

**Glasgow Coma Scale**

| **Parameter** | **Response** | **Score** | **Assessment** |
| --- | --- | --- | --- |
| **Eye Opening** | Spontaneous | 4 | Spontaneous eye opening receives the highest score for this parameter. Eye opening is considered "to speech" when it occurs in response to any verbal stimulus—spoken or shouted—regardless of whether the patient is specifically instructed to open their eyes. When assessing eye opening in response to pain, stimuli should be applied to an extremity. Central stimuli such as supraorbital pressure or pressure at the mandibular angle may elicit facial reflexes that mimic voluntary eye closure, potentially leading to misinterpretation. |
|  | To speech | 3 |  |
|  | To pain | 2 |  |
|  | No response | 1 |  |
| **Verbal Response** | Oriented | 5 | An oriented verbal response indicates that the patient is aware of their identity, location, situation, and time—demonstrated by their ability to state who they are, why they are there, and the current year. A confused verbal response occurs when the patient can engage in conversation and answer questions, but their replies reveal disorientation or confusion. An inappropriate verbal response is characterized by disorganized speech, often consisting of random, shouted, or profane words that do not allow for meaningful interaction. Vocalizations such as moaning or groaning without recognizable words are classified simply as sounds. |
|  | Confused | 4 |  |
|  | Inappropriate words | 3 |  |
|  | Incomprehensible sounds | 2 |  |
|  | No response | 1 |  |
| **Motor Response** | Obeys commands | 6 | When assessing motor response and the ability to follow commands in a patient with traumatic brain injury, commands should be simple and clearly stated, avoiding misinterpretation of primitive reflexes such as the grasp reflex or involuntary postural adjustments as intentional responses. If there is no observable response to verbal commands, a sustained painful stimulus should be applied to the nail bed of each upper extremity to elicit the maximal motor response. If the patient responds with an extensor posture, this is considered a decerebrate response. If a flexor posture is observed, a central painful stimulus should then be applied to the head or neck to further assess the type of response. Purposeful movement toward the stimulus indicates localization; a rapid withdrawal movement, typically with shoulder abduction, indicates withdrawal; and a posture characterized by shoulder adduction with flexion of the upper limbs and extension of the lower limbs indicates decorticate posturing. If no motor response is present, spinal cord injury or vertebral translocation should be suspected. In such cases, command following should be assessed using cranial nerve–innervated muscles, such as facial expressions or tongue movements. |
|  | Localizes pain | 5 |  |
|  | Withdraws pain | 4 |  |
|  | Decorticate posturing | 3 |  |
|  | Descerebrate posturing | 2 |  |
|  | No response | 1 |  |

**APPENDIX B.** Prehospital and in-hospital interventions for the management of traumatic thoracic life-threatening conditions.

| **PREHOSPITAL INTERVENTIONS** | |
| --- | --- |
| **Condition** | **Interventions** |
| Cardiac tamponade | Maintain BP using crystalloid fluids boluses as needed, and initiate rapid transfer to a facility with surgical capabilities, ensuring that the receiving center is notified prior to the patient’s arrival. |
| Tension pneumothorax | Perform needle decompression using the largest available bore needle. Insert the needle perpendicular to the chest wall at the fourth or fifth intercostal space, within the safe triangle—bounded anteriorly by the lateral edge of the pectoralis major muscle and posteriorly by the medial border of the serratus anterior—ensuring placement above the superior margin of the lower rib to avoid neurovascular injury. Advance the needle fully until air is heard escaping, indicating entry into the pleural space. Withdraw the needle, leaving the catheter in place and connected to a one-way valve (e.g., Heimlich valve) to allow ongoing decompression. Then, initiate rapid transfer to a facility with surgical capabilities, ensuring that the receiving center is notified prior to the patient’s arrival. |
| Open pneumothorax | Apply a one-way valve dressing. If a commercial chest seal is not available, a makeshift valve can be created by cutting a sterile square from a Viaflex (IV fluid) bag and taping it securely on three sides over the wound, leaving one side open to function as a flutter valve. This dressing must be monitored closely for signs of tension pneumothorax. Then, initiate rapid transfer to a facility with surgical capabilities, ensuring that the receiving center is notified prior to the patient’s arrival. |
| Massive hemothorax | Administer supplemental oxygen using the highest-flow device available. Maintain BP with boluses of crystalloid fluids as needed. Initiate rapid transfer to a facility with surgical capabilities, ensuring that the receiving center is notified in advance of the patient’s arrival. |
| Flail chest | Administer supplemental oxygen using the highest-flow device available. Provide the most effective pain control available, at the provider’s discretion. Initiate rapid transfer to a facility with surgical capabilities, ensuring that the receiving center is notified in advance of the patient’s arrival. |

| **IN-HOSPITAL INTERVENTIONS** | |
| --- | --- |
| **Condition** | **Interventions** |
| Cardiac tamponade | Maintain BP with crystalloid fluid or blood products boluses as needed, and urgently obtain an Acute Care Surgery consult for definitive management. |
| Tension pneumothorax | Perform needle decompression using the largest available bore needle. Insert the needle perpendicular to the chest wall at the fourth or fifth intercostal space, within the safe triangle—bounded anteriorly by the lateral edge of the pectoralis major muscle and posteriorly by the medial border of the serratus anterior—ensuring placement above the superior margin of the lower rib to avoid neurovascular injury. Advance the needle fully until air is heard escaping, indicating entry into the pleural space. Withdraw the needle, leaving the catheter in place and connected to a one-way valve (e.g., Heimlich valve) to allow ongoing decompression. Then, according to institutional protocol, place a chest tube/pigtail catheter yourself or request an Acute Care Surgery consultation at the same decompression site. |
| Open pneumothorax | Apply a one-way valve dressing. If a commercial chest seal is unavailable, create a makeshift valve by cutting a sterile square from a Viaflex (IV fluid) bag and taping it securely on three sides over the wound, leaving one side open to function as a flutter valve. Then, either place a chest tube or pigtail catheter yourself, or request an Acute Care Surgery consultation for placement within the safe triangle—bounded anteriorly by the lateral edge of the pectoralis major muscle and posteriorly by the medial border of the serratus anterior—ensuring insertion above the superior margin of the lower rib to avoid neurovascular injury. Finally, suture the communicating wound. |
| Massive hemothorax | Administer supplemental oxygen using the highest-flow device available. Maintain blood pressure with boluses of crystalloid fluids or blood products as needed. Then, either place a chest tube yourself or request an acute care surgery consultation for placement within the safe triangle—bounded anteriorly by the lateral edge of the pectoralis major muscle and posteriorly by the medial border of the serratus anterior—ensuring insertion above the superior margin of the lower rib to avoid neurovascular injury. If chest tube output exceeds 1,500 mL immediately upon placement or remains greater than 200 mL per hour for three consecutive hours, the patient must be taken to the operating room by Acute Care Surgery for definitive hemorrhage control. |
| Flail chest | Administer supplemental oxygen using the highest-flow device available. Provide optimal pain control at the provider’s discretion, and obtain an Acute Care Surgery or Thoracic Surgery consultation for definitive management. |

**APPENDIX C.** Trauma Associated Severe Hemorrhage [TASH] Score and Assessment of Blood Consumption [ABC] Score

| **ABC Score** | **TASH Score** |
| --- | --- |
| Penetrating torso injury  HR ≥ 120 bpm  SBP ≤ 90 mmHg  Positive FAST exam | **Hemoglobin (g/dL):**  < 7 (8 pts)  7-9 (6 pts)  9-10 (4 pts)  10-11 (3 pts)  11-12 (2 pts)  **Base Excess (mmol/L)**  < -10 (4 pts)  -10 to -6 (3 pts)  -6 to -2 (1 pt)  **SBP (mmHg)**  < 100 (4 pts)  100 - 120 (1 pt)  **HR**  > 120 bpm (2 pts)  Positive FAST exam (3 pts)  Clinically unstable pelvis fracture (6 pts)  Open/dislocated femoral fracture (3 pts)  Male sex (1 pt) |
| **Interpretation:**  Each item equals 1 point. A score ≥ 2 suggests a higher likelihood of requiring massive transfusion. | **Interpretation:**  The total TASH score, calculated as the sum of individual item points, correlates with the probability of requiring a massive transfusion. A higher score indicates a greater likelihood. A commonly accepted threshold is a score of ≥16, at which point activation of a massive transfusion protocol should be strongly considered. |
