# Supplementary material for: Second edition of the recommendations from the Colombian consensus committee for the management of traumatic brain injury in the prehospital setting, emergency department, surgery, and intensive care (Beyond one option for treatment of traumatic brain injury: A stratified protocol [BOOTStraP])
Source: Brain Spine. 2026 Apr 12;6:106046. doi: 10.1016/j.bas.2026.106046 (PMC13101607; doi:10.1016/j.bas.2026.106046)
Supplement: Multimedia component 4 [file mmc4.docx]

**Supplementary material S4**

**BOOTStraP second edition Implementation Checklist**

*For use by healthcare institutions adopting the BOOTStraP protocol*

**Institution Information**

| **Institution name:** |  |
| --- | --- |
| **City / Department:** |  |
| **Level of complexity:** |  |
| **Responsible coordinator:** |  |
| **Implementation date:** |  |

**Checklist**

| **#** | **Item** | **☐ Done** |
| --- | --- | --- |
| **1. Staff Training — Color-Coded Algorithm Interpretation** | | |
| **1.1** | Identify designated BOOTStraP trainer(s) for each clinical area (prehospital, emergency, surgery, ICU) | ☐ |
| **1.2** | Conduct initial training session on color-coded resource stratification (red/yellow/green) for all clinical staff involved in TBI care | ☐ |
| **1.3** | Train staff on algorithm navigation: selecting the appropriate algorithm based on resource availability and care phase | ☐ |
| **1.4** | Emphasize that algorithms are decision-support tools requiring clinical judgment, not prescriptive protocols | ☐ |
| **1.5** | Conduct refresher training sessions every 6 months | ☐ |
| **1.6** | Maintain training log with dates, attendees, and competency assessment results | ☐ |
| **2. Medication Availability Audit** | | |
| **2.1** | Verify availability of tranexamic acid (TXA) in prehospital and emergency units | ☐ |
| **2.2** | Verify availability of hypertonic saline (HTS 3% and/or 7.5%) in emergency and ICU | ☐ |
| **2.3** | Verify availability of RSI medications: ketamine, midazolam, succinylcholine, rocuronium | ☐ |
| **2.4** | Verify availability of vasopressors: noradrenaline, adrenaline | ☐ |
| **2.5** | Verify availability of antiepileptic agents: levetiracetam or phenytoin | ☐ |
| **2.6** | Create a BOOTStraP core medications list and integrate into institutional formulary | ☐ |
| **2.7** | Establish alert system for critical medication shortages affecting BOOTStraP protocols | ☐ |
| **3. Telemedicine Connectivity** | | |
| **3.1** | Verify telemedicine infrastructure availability (internet, device, platform) in emergency and prehospital units | ☐ |
| **3.2** | Establish formal teleconsultation agreement with a neurosurgical referral center | ☐ |
| **3.3** | Define and document the neurosurgical teleconsultation protocol: contact, response time, documentation | ☐ |
| **3.4** | Conduct a test teleconsultation drill before clinical implementation | ☐ |
| **3.5** | Define backup communication protocol when primary telemedicine system fails | ☐ |
| **4. Non-Invasive Neuromonitoring Equipment** | | |
| **4.1** | Verify availability of ONSD measurement capability: linear ultrasound probe (7.5-10 MHz) and trained operator | ☐ |
| **4.2** | Verify availability of transcranial Doppler (TCD) for pulsatility index and cerebral autoregulation assessment | ☐ |
| **4.3** | Verify availability of automated pupillometry (NPi device) or establish structured manual pupil assessment protocol | ☐ |
| **4.4** | Train at least one operator per shift in ONSD measurement technique | ☐ |
| **4.5** | Integrate non-invasive neuromonitoring results into clinical decision-making using BOOTStraP Table 11 thresholds | ☐ |
| **4.6** | Establish quality control protocol for non-invasive neuromonitoring measurements | ☐ |
| **5. Serum Biomarkers (GFAP / UCH-L1)** | | |
| **5.1** | Verify laboratory availability of GFAP and/or UCH-L1 assays (Abbott i-STAT, Alinity, or equivalent platform) | ☐ |
| **5.2** | Confirm sample type required (plasma from centrifuged whole blood) and processing time | ☐ |
| **5.3** | Train clinical staff on time-dependent interpretation: thresholds (GFAP >30 pg/mL, UCH-L1 >360 pg/mL) valid only within 12 hours of injury | ☐ |
| **5.4** | Establish result communication protocol from laboratory to emergency team within defined timeframe | ☐ |
| **5.5** | Document that biomarker results are adjuncts to clinical judgment and CT imaging, not substitutes | ☐ |
| **5.6** | Conduct cost-effectiveness analysis for institutional biomarker implementation | ☐ |
| **6. Inter-Facility Transfer Protocol** | | |
| **6.1** | Identify and document referral centers with neurosurgical capabilities and their contact information | ☐ |
| **6.2** | Establish written inter-facility transfer protocol: decision criteria, transport mode, accompanying personnel | ☐ |
| **6.3** | Verify transport availability: ground ambulance and air transport if referral center >150 km | ☐ |
| **6.4** | Define neuroprotection bundle to maintain during transfer (BOOTStraP Table 12) | ☐ |
| **6.5** | Conduct simulation drill for TBI patient transfer at least once per year | ☐ |
| **7. Outcome Tracking** | | |
| **7.1** | Designate responsible person for data collection and registry | ☐ |
| **7.2** | Implement minimum data collection: GCS on admission, resource level used, algorithm applied, disposition | ☐ |
| **7.3** | Assess neurological outcome using GOS-E at hospital discharge | ☐ |
| **7.4** | Assess neurological outcome using GOS-E at 30 days post-injury | ☐ |
| **7.5** | Submit anonymized data to BOOTStraP national registry (when available) | ☐ |
| **7.6** | Perform institutional review of BOOTStraP outcomes every 6 months | ☐ |
| **8. Local Adaptation Documentation** | | |
| **8.1** | Document local resource level for each care phase using BOOTStraP Table 1 | ☐ |
| **8.2** | Identify which algorithms apply to your institution based on available resources | ☐ |
| **8.3** | Document local regulatory constraints affecting BOOTStraP recommendations | ☐ |
| **8.4** | Adapt color-coded resource levels to local context and document deviations from standard protocol | ☐ |
| **8.5** | Submit local adaptation documentation to BOOTStraP coordinating center | ☐ |

**Abbreviations**

GCS = Glasgow Coma Scale; GFAP = Glial Fibrillary Acidic Protein; GOS-E = Glasgow Outcome Scale Extended; HTS = Hypertonic Saline; ICU = Intensive Care Unit; ICP = Intracranial Pressure; NPi = Neurological Pupil Index; ONSD = Optic Nerve Sheath Diameter; RSI = Rapid Sequence Intubation; TBI = Traumatic Brain Injury; TCD = Transcranial Doppler; TXA = Tranexamic Acid; UCH-L1 = Ubiquitin Carboxy-Terminal Hydrolase L1; ED = Emergency Department.
